# Doxycycline-dependent Cas9-expressing pig resources for conditional in vivo gene nullification and activation

**DOI:** 10.1186/s13059-023-02851-x

**Published:** 2023-01-17

**Authors:** Qin Jin, Xiaoyi Liu, Zhenpeng Zhuang, Jiayuan Huang, Shixue Gou, Hui Shi, Yu Zhao, Zhen Ouyang, Zhaoming Liu, Lei Li, Junjie Mao, Weikai Ge, Fangbing Chen, Manya Yu, Yezhi Guan, Yinghua Ye, Chengcheng Tang, Ren Huang, Kepin Wang, Liangxue Lai

**Affiliations:** 1 China-New Zealand Joint Laboratory on Biomedicine and Health, CAS Key Laboratory of Regenerative Biology, Guangdong Provincial Key Laboratory of Stem Cell and Regenerative Medicine, Centre for Regenerative Medicine and Health, Hong Kong Institute of Science and Innovation, Guangzhou Institutes of Biomedicine and Health, Chinese Academy of Sciences, Guangzhou, 510530 China; 2 Research Unit of Generation of Large Animal Disease Models, Chinese Academy of Medical Sciences (2019RU015), Guangzhou, 510530 China; 3 Sanya Institute of Swine Resource, Hainan Provincial Research Centre of Laboratory Animals, Sanya, 572000 China; 4 University of Chinese Academy of Sciences, Beijing, 100049 China; 5 Guangdong Provincial Key Laboratory of Laboratory Animals, Guangdong Laboratory Animals Monitoring Institute, Guangzhou, 510633 China; 6 Guangdong Provincial Key Laboratory of Large Animal models for Biomedicine, School of Biotechnology and Health Science, Wuyi University, Jiangmen, 529020 China; 7 Joint School of Life Sciences, Guangzhou Institutes of Biomedicine and Health, Chinese Academy of Sciences, Guangzhou Medical University, Guangzhou, 510530 China

## Abstract

**Background:**

CRISPR-based toolkits have dramatically increased the ease of genome and epigenome editing. SpCas9 is the most widely used nuclease. However, the difficulty of delivering SpCas9 and inability to modulate its expression in vivo hinder its widespread adoption in large animals.

**Results:**

Here, to circumvent these obstacles, a doxycycline-inducible SpCas9-expressing (DIC) pig model was generated by precise knock-in of the binary tetracycline-inducible expression elements into the *Rosa26* and *Hipp11* loci, respectively. With this pig model, in vivo and/or in vitro genome and epigenome editing could be easily realized. On the basis of the DIC system, a convenient Cas9-based conditional knockout strategy was devised through controlling the expression of rtTA component by tissue-specific promoter, which allows the one-step generation of germline-inherited pigs enabling in vivo spatiotemporal control of gene function under simple chemical induction. To validate the feasibility of in vivo gene mutation with DIC pigs, primary and metastatic pancreatic ductal adenocarcinoma was developed by delivering a single AAV6 vector containing *TP53*-sgRNA, *LKB1*-sgRNA, and mutant human *KRAS* gene into the adult pancreases.

**Conclusions:**

Together, these results suggest that DIC pig resources will provide a powerful tool for conditional in vivo genome and epigenome modification for fundamental and applied research.

**Supplementary Information:**

The online version contains supplementary material available at 10.1186/s13059-023-02851-x.

## Background

Pigs are not only important domestic animals providing food for human, but also desirable models for biomedical discovery because they share many similarities with humans in terms of immune system, anatomy, physiology, organ size, and metabolism [1]. Although gene-edited (GE) pig models are not as universal as rodent models, an increasing number of GE pigs of critical importance for farm product [2, 3], xenotransplantation [4–6], and human disease modeling [7–9] have been generated to date.

At present, GE pigs are mainly generated through direct embryonic microinjection of custom endonuclease-based components or gene editing in somatic cells followed by somatic cell nuclear transfer (SCNT) cloning, both of which have long been extremely laborious, inefficient, and time consuming [10]. Specifically, the direct embryonic microinjection approach cannot assure uniform genotypes and thus results in mosaic individuals, requiring long inter-crossing programs to obtain animals with desirable genotypes, and hardly enables precise gene knock-in via homology-directed repair (HDR). Alternatively, uniform genotypes or precise knock-in can be fulfilled through the SCNT approach. However, simultaneously multiple knock-in seems impossible due to the low efficiency of HDR. Therefore, obtaining GE pig lines with precise multiplex gene editing, especially spatiotemporally restricted genetic modification consisting of widely used Cre-*lox*P system with multiplex precise knock-in still remains a huge challenge.

To address the issues above, direct in vivo genome editing through delivering the expression vectors of Cas9 and sgRNAs into selected tissues of adult mice had been performed [11–14]. However, the overall editing efficiency was low because this approach was mediated by lentivirus or adeno-associated virus (AAV), which is inefficient to produce due to the large size of the SpCas9 gene (∼4.2 kb) [15]. To overcome this problem, a mouse model in which the Cre-dependent Cas9-expressing cassette was specifically inserted into the *Rosa26* locus was generated [16]. Subsequently, Cre and sgRNAs targeting genes of interest were introduced to specific somatic cell types to allow in vivo genome editing to be conveniently and efficiently performed, which circumvents the requirement to generate new germline-modified founder lines [16–18]. This is an ideal alternative especially when applied to large animals such as pigs to shorten production time and save costs. More recently, the generation of constitutively Cas9-expressing animal models, such as *Rosa26*-Cas9 transgenic pigs, dramatically reduced the payload size of viral vectors and the number of components required to be delivered thereby promoting in vivo gene editing [19] but still unable to achieve time or spatial controlled expression of SpCas9 protein in vivo. Additionally, genomic damages [20, 21], off-target effects [22, 23], and immunological clearance responses [24] caused by uncontrollable Cas9 expression, will hinder the application of animal models with this constitutively Cas9-expressing system, which is also not avoidable for inducible expression systems based on the Cre-recombinase system, in which sustainable constitutive Cas9 expression exist after activation [16, 25]. Furthermore, Cre-recombinase was confirmed to negatively affect porcine embryonic development [26] and to be potentially genotoxic with pseudo recombination sites in mammalian genomes [27].

Tetracycline-inducible over-expression (Tet-On) system is one of the most prominent and widely accepted inducible systems, which has been most extensively employed in transgenic mouse modeling [28]. The Tet-On system uses a reverse tetracycline transactivator (rtTA) protein that binds to the tetracycline-response element (TRE) to activate the transcription of foreign gene of interest in the presence of doxycycline (Dox). By using the tetracycline-inducible system with a flexibly temporal regulation feature, Cas9 expression can be repeatedly turned on or off under Dox induction. Moreover, the chemical inducer, Dox, is easier to access and deliver to targeted sites than the Cre-recombinase.

In this study, through the precise knock-in of the binary Tet-On elements, rtTA-expressing cassette and TRE-controlled SpCas9-T2A-tdTomato cassette, into the *Rosa26* [29] and *Hipp11* [30] loci, respectively, we attempted to generate a Dox-inducible Cas9-expressing (DIC) pig model that allows flexible temporal control of SpCas9 activity in pigs by simple chemical induction in vitro and in vivo. With this pig model, in vitro and/or in vivo gene editing and chromosome engineering for loss of function and in vitro regulation of endogenous target gene for gain of function could be realized by simply delivering the sgRNAs or transcriptional activation complexes combined with modified dead guide RNAs into porcine fetal fibroblasts (PFFs) and/or specific tissues and following Dox induction. Besides, on the basis of PFFs carrying TRE-controlled SpCas9-T2A-tdtomato allele, the 2A-rtTA-polyA cassette and U6-sgRNA-expressing cassette were inserted to replace the stop codon of tissue-specific expressing genes, which could allow one-step generation of germline inherited pigs enabling spatiotemporal control of gene function in vivo under chemical induction. Given the remarkable benefits of cancer modeling mediated by in vivo gene mutation on large animals with a human-closer competent immune system compared with rodents, primary and metastatic pancreatic ductal adenocarcinoma (PDAC) pig model was developed by delivering *TP53*-sgRNA, *LKB1*-sgRNA, and mutant human *KRAS* gene into the adult pancreases of DIC pigs and following Dox induction. The DIC pig model overcomes the in vivo delivery challenge of Cas9 protein, avoids the side effects of constitutive Cas9 expression, and eliminates the potential Cre-mediated genotoxicity, which will provide a powerful and flexible platform for conditional in vivo genome and epigenome editing for biomedical and agricultural applications.

## Results

### Generation of a DIC pig line

**Fig. 1:**
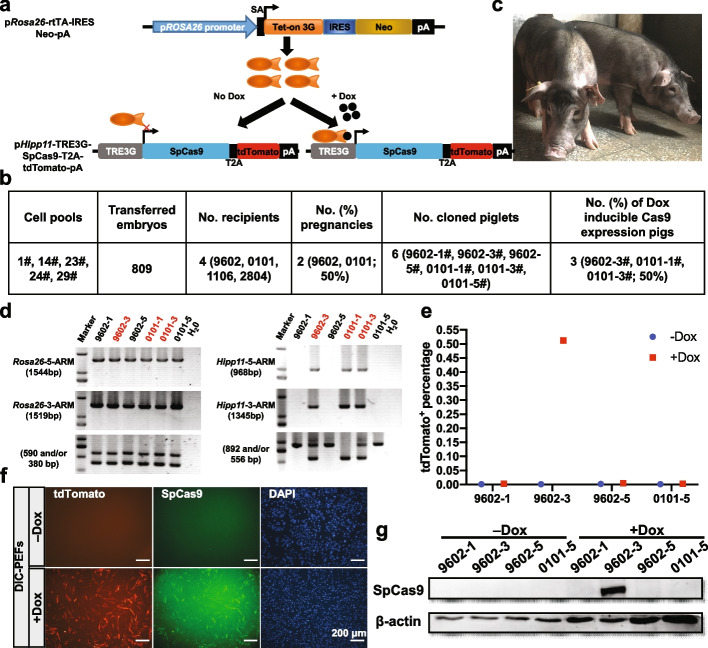
Generation of the Dox-inducible SpCas9-expressing (DIC) pig line. **a** Schematic of the gene targeting strategy and SpCas9 expression induced by Dox. The rtTA is contained within the p*Rosa26* locus. The TRE3G-controlled SpCas9-T2A-tdTomato-expressing cassette is contained within the p*Hipp11* locus. SpCas9 and tdTomato expression is tightly regulated by Dox. SA, splice acceptor. **b** Summary of SCNT experiments for generating DIC pigs. **c** Representative photo of the two DIC founders, 10-month-old 9602-3# (left) and 0101-1# (right). Both are male. **d** PCR analysis confirmed the correct homologous recombination at the p*Rosa26* and *Hipp11* loci in cloned piglets. **e**–**g** Assay of the expression level of SpCas9 and tdTomato protein, induced by Dox, in fibroblasts isolated from the ear tissues of cloned piglets shown in (**d**). PEFs were treated with or without Dox, and then the Dox-induced tdTomato expression is assayed by FACS analysis (**e**). Immunofluorescence staining (**f**) and Western blotting (**g**) were used to further validate the uniform expression of SpCas9 and tdTomato protein in DIC PEFs with Dox induction. Scale bars, 200 μm

To flexibly induce the expression of SpCas9 in pigs, a binary third-generation tetracycline-inducible (Tet-On 3G) system, consisting of the rtTA and TRE3G-controlled SpCas9-T2A-tdTomato expressing cassette, was used (Fig. 1a). The PFFs carrying rtTA-expressing cassette in the porcine *Rosa26* (p*Rosa26*) locus (p*Rosa26*-rtTA PFFs), but not including TRE3G-tdTomato-expressing cassette, were isolated from crossbreeding our previously generated Dox-inducible tdTomato-expressing pigs [31] (Additional file 1: Fig. S1a). A targeting vector containing TRE3G-driving SpCas9-T2A-tdTomato-expressing cassette and puromycin resistance cassette, flanked by 911 bp 5′ arm and 1088 bp 3′ arm from the porcine *Hipp11* (p*Hipp11*) locus, another confirmed safe harbor site [30], were designed (Additional file 1: Fig. S1b). With this vector, the utility of the 2A peptide could assure the uniform expression of SpCas9 and tdTomato, the single-cell-derived positive colonies could be selected by puromycin treatment, and the expression of SpCas9 could be displayed by the indicator of tdTomato. TRE3G-controlled SpCas9 vectors were inserted into the p*Hipp11* locus in p*Rosa26*-rtTA PFFs by CRISPR/Cpf1-mediated HDR (Additional file 1: Fig. S1b). A total of 44 single-cell-derived colonies were picked after about 10-day puromycin selection. For these colonies, 38 were successfully expanded in 24-well plates and further screened by 5′- and 3′- junction fragment PCR. The PCR results showed that 22 colonies carried TRE3G-SpCas9-T2A-tdTomato elements in the p*Hipp11* locus (Additional file 1: Fig. S1c, d). Two representative cell colonies (1# and 23#) and re-constructed SCNT embryos generated by using these cells as donors were treated with Dox for 3 days. The tdTomato fluorescence was observed in Dox-treated cell colonies and re-constructed embryos under an inverted fluorescence microscope, whereas no tdTomato fluorescence was found without Dox induction (Additional file 1: Fig. S1e, f). When reconstructed embryos were cultured for 6 days, almost no difference of blastocyst rates between Dox-treated (19.5%, 40/205) and Dox-untreated (20.4%, 23/113) groups was observed, while the previous report showed that zygote injection of RNA encoding Cre recombinase resulted in reduction of blastocyst rate [26], indicating that Dox-dependent SpCas9 expression may have less negative impact on porcine embryo development than Cre recombinase expression. These results suggested that the established DIC system in pigs could result in Dox-dependent SpCas9 expression at the cellular and embryonic level.

Next, five positive cell colonies (1#, 14#, 23#, 24#, and 29#) were pooled together and used as the nuclear donors for SCNT. A total of 809 reconstructed embryos were generated and then surgically transferred into four surrogate mothers (9602, 0101, 1106, and 2804). Two surrogates (50%, 2/4, 9602 and 0101) were confirmed to be pregnant and developed to full term. After about 113 days of gestation, six male cloned piglets (9602-1#, 9602-3#, 9602-5#, 0101-1#, 0101-3#, and 0101-5#) were delivered naturally (Fig. 1b, c). The 5′- and 3′-junction fragment PCR showed that two piglets (9602-3# and 0101-3#) carried heterozygous TRE3G-SpCas9-T2A-tdTomato-expressing cassette and one piglet (0101-1#) harbored homozygous TRE3G-SpCas9-T2A-tdTomato-expressing cassette in the p*Hipp11* locus (Fig. 1d; Additional file 1: Fig. S1a, b). All six cloned pigs carried heterozygous rtTA-expressing cassette in the p*Rosa26* locus. Except for 0101-3#, which was born weak and died early, the other two piglets (9602-3# and 0101-1#) were healthy and grew up to adult age without overt abnormalities (Fig. 1c). The low cloning efficiency and the perinatal death might be due to overall inefficient SCNT procedures as that previously reported [32], rather than genetic modification of donor cells per se. The porcine ear fibroblasts (PEFs) were isolated from the ear tissues of the cloned piglets and cultured in the medium with or without Dox. The tdTomato fluorescence was observed under an inverted fluorescence microscope (Fig. 1f), and approximately 51.2% DIC-PEFs were tdTomato positive by fluorescence-activated cell sorting (FACS) analysis (Fig. 1e). No tdTomato fluorescence was observed in DIC-PEFs without Dox treatment and p*Rosa26*-rtTA PEFs with and without Dox treatment. SpCas9 proteins were further assayed by immunofluorescence staining and Western blot. The results showed that SpCas9 and tdTomato proteins were uniformly expressed in DIC-PEFs with Dox induction (Fig. 1f, g). These results suggested that the expression of SpCas9 and tdTomato was consistent and depended on Dox presence in pigs in vitro.

By crossing the two male DIC founders (9602-3# and 0101-1#) with six wild-type sows, 39 of F1 pigs were delivered. Of these offspring, 11 simultaneously carried rtTA-expressing cassette and TRE3G-SpCas9-T2A-tdTomato-expressing cassette in the p*Rosa26* and p*Hipp11* loci, respectively (Additional file 2: Table S1). The established DIC transgenic pig line was used for further study.

### Establishment of Dox administration protocol for inducing SpCas9 expression and assessment of SpCas9 expression level in different organs of DIC pigs

**Fig. 2:**
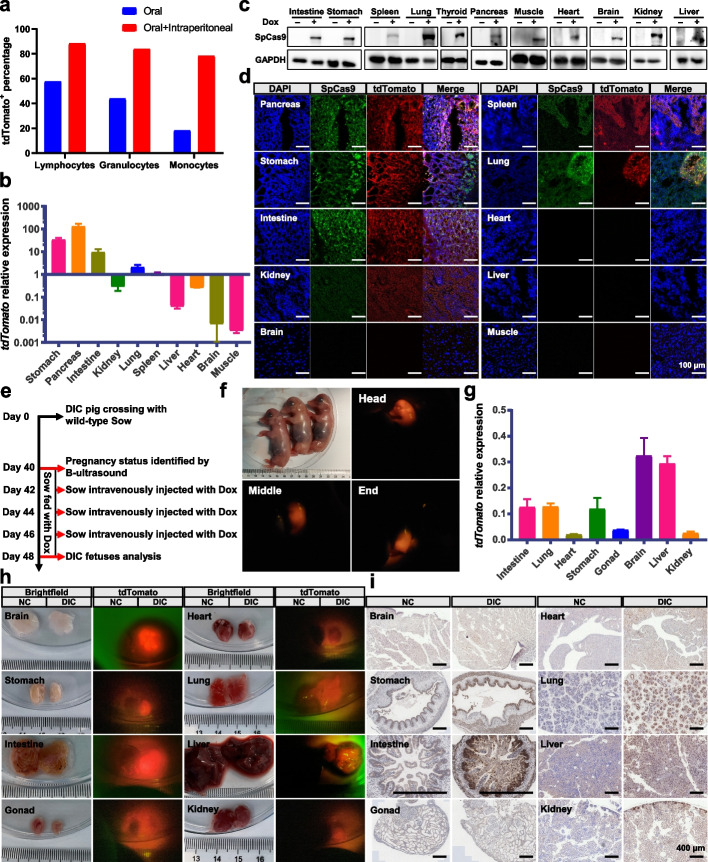
Establishment of Dox administration protocol for inducing SpCas9 expression and assessment of SpCas9 expression levels in different organs. **a** FACS analysis of the tdTomato expression of PBMCs in postnatal DIC pigs treated with Dox. Blue indicates oral administration (50 mg/kg/day) for a week; red indicates oral administration (50 mg/kg/day) for a week combined with three intraperitoneal injections (50 mg/kg) every other day. Age-matched wild-type pigs treated with Dox via the same administration strategy were used as the negative control. **b**–**d** Validation of the SpCas9 expression in multiple organs of postnatal DIC pigs treated with Dox via oral administration and intraperitoneal injection by Q-PCR analysis, normalized to the *GAPDH.* Data were collected from three independent experiments (*n* = 3) and presented as mean ± SEM (**b**), Western blotting (**c**), and immunofluorescence staining (**d**). Age-matched pigs not treated with Dox were used as the negative control. Scale bars, 100 μm. **e** Schematic of Dox administration strategy for pregnant sow. The sow at 42-day gestation was fed with Dox (50 mg/kg/day) for 1 week combined with three intravenous injections (50 mg/kg) every other day. At 48 days, DIC fetuses were retrieved for further study. **f** Fluorescent images of the tdTomato expression of E48 porcine fetuses. The porcine fetus in the middle was a DIC individual, and the rest were wild-type individuals. **g** Q-PCR results of the tdTomato expression in multiple organs of DIC porcine fetuses (*n* = 3), normalized to the *GAPDH*, presented as mean ± SEM. **h**–**i** Characterization of the SpCas9 expression in multiple organs of DIC porcine fetuses with Dox induction by fluorescence observation (**h**) and immunohistochemical staining (**i**)

To establish Dox administration protocol for inducing SpCas9 expression in vivo, we first treated the DIC pigs with Dox via oral administration. When DIC piglets were orally administrated with low dosage of Dox (25 mg/kg/day), weak tdTomato fluorescence was observed in most organs through using goggles in a dark room with appropriate excitation and emission filters. After oral administration of high dosage of Dox (50 mg/kg/day), tdTomato fluorescence was obviously increased (Additional file 1: Fig. S2). These results indicated that the expression level of SpCas9 in vivo depended on Dox dosage. However, only about 20% of peripheral blood mononuclear cells (PBMCs) (18.1% for monocytes, 43.8% for granulocytes, and 57.5% for lymphocytes) were tdTomato positive after one-week oral administration with high dosage (Fig. 2a). Next, we further verified whether oral administration combined with intraperitoneal injection could improve the induction efficiency in vivo. The FACS results of peripheral blood showed that more than 80% PBMCs (78.2% for monocytes, 83.7% for granulocytes, and 88.3% for lymphocytes) expressed the tdTomato fluorescence, suggesting that oral administration combined with intraperitoneal injection of Dox could significantly increase the induction efficiency (Fig. 2a). Immunohistochemistry (IHC) staining results also confirmed that a wide range of induction of SpCas9 expression in many tissues and organs, when compared with oral administration of Dox only (Additional file 1: Fig. S3). Therefore, oral administration combined with intraperitoneal injection was selected as the standard method to deliver Dox in the following experiments.

Next, we detected the expression levels of SpCas9 in different organs after treatment with Dox by quantitative PCR (Q-PCR), Western blotting, and immunofluorescence staining. These results showed that the SpCas9 protein were expressed in all assessed organs but displayed different levels in different organs (Fig. 2b, c, d). Among the tested organs, the pancreas, stomach, intestine, lung, and spleen showed high expression levels of SpCas9, whereas the brain, skeletal muscle, liver, and heart showed low expression levels. The relative low expression of SpCas9 in the brain and liver was possibly due to Dox inaccessibility and degradation, respectively. For the heart and skeletal muscle, possibly the low rtTA expression due to the weak activity of endogenous Rosa26 promoter in these organs, the same as previously reported in mice [33], might account for the low SpCas9 expression. These results indicated that broad and tightly controlled SpCas9 expression was achieved in DIC pigs in vivo through Dox treatment.

Time-controlled SpCas9 expression at the embryonic stage is very important for lineage tracing and embryonic conditional gene knockout. Therefore, we next verified whether the expression of SpCas9 could be efficiently induced at the embryonic development stage by treating pregnant sows with Dox. The DIC founder, 0101-1#, was cross-mated with one wild-type sow, and the pregnancy status was confirmed by B-ultrasound. The sow at 42 days of gestation was fed with Dox for 6 days combined with three intravenous injections every other day (Fig. 2e). The sow at 48 days of gestation was sacrificed to retrieve DIC fetuses. Twelve fetuses were obtained, and six were observed with high level of tdTomato fluorescence in the whole body by using goggles with appropriate excitation and emission filters (Fig. 2f). The brain, heart, stomach, lung, intestine, liver, and gonad were collected for further analysis. Obvious tdTomato fluorescence was observed in all collected organs (Fig. 2h). Q-PCR and IHC staining results also suggested broad SpCas9 expression in DIC fetuses with Dox induction (Fig. 2g, i). Notably, the fetal brain and liver expressed high levels of SpCas9 and tdTomato, which were different from adult DIC pigs in vivo, possibly due to the absence of the blood-brain barrier and less developed liver function in 48-day-old porcine fetuses. These results confirmed that time-dependent tight in vivo control of SpCas9 expression can be broadly achieved in most organs of fetuses through treatment of pregnant DIC sows with Dox.

### Validation of the DNA cleavage activity of Dox-induced SpCas9 protein for genomic editing ex vivo

To validate the DNA cleavage activity of Dox-induced SpCas9 protein ex vivo, DIC-PFFs were isolated from 35-day-old fetuses retrieved from a pregnant wild-type sow mated with the 0101-1# DIC founder. Six fetuses were obtained, and two fetuses (DIC-F1-3 and DIC-F1-4) simultaneously carried heterozygous knock-in alleles at the p*Rosa26* and p*Hipp11* loci, respectively (Additional file 1: Fig. S4a). The remaining four fetuses (DIC-F1-1, DIC-F1-2, DIC-F1-5, and DIC-F1-6) only carried heterozygous TRE3G-SpCas9-T2A-tdTomato-expressing allele at the p*Hipp11* locus. The Western blotting and immunofluorescence staining results showed that the expression of SpCas9 was tightly controlled by Dox in DIC-F1-PFFs (Additional file 1: Fig. S4b-d). Through continuous stimulation with Dox, the percentage of tdTomato-positive PFFs gradually increased from day 1 to day 6. Following withdrawal of Dox, the percentage of tdTomato-positive PFFs gradually decreased (Additional file 1: Fig. S4e, f).

**Fig. 3:**
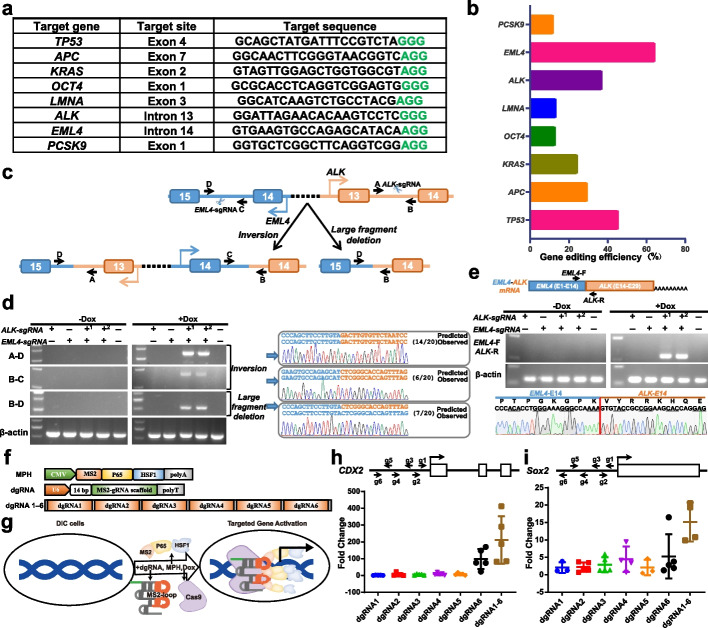
Targeted genome editing and transcriptional activation of porcine endogenous genes using the DIC system. **a** Target genes and sgRNA sequences used to detect editing efficiency. **b** Efficiency of genome editing for target genes shown in (**a**) after transfecting corresponding sgRNA into DIC PFFs. **c** Schematic of the chromosome engineering events in the porcine *ALK* and *EML4* loci after simultaneously transfecting ALK-sgRNA and EML4-sgRNA into DIC PFFs. **d** PCR analysis and Sanger sequencing of chromosomal inversion and large fragment deletion between *ALK* and *EML4* loci. **e** RT-PCR analysis and Sanger sequencing of EML4-ALK fusion transcripts resulting from chromosomal inversion between *ALK* and *EML4* loci. In **d** and **e**, “+1” group refers to the simultaneous transfection of two plasmids containing *ALK*-sgRNA and *EML4*-sgRNA, respectively; “+2” group refers to transfection of a single plasmid containing pre-tRNA-linked *ALK*- and *EML4*-sgRNAs. **f** Schematic of transcriptional activation constructs. MPH, MS2-P65-HSF1 complexes controlled by CMV promoter. dgRNA, nuclease-dead single guide RNA, driven by U6 promoter, harboring MS2-loop to recruit MS2-P65-HSF1 complexes and a 14-bp target sequence, which inactivates SpCas9. dgRNA 1-6, representing six dgRNAs driven by its own U6 promoter in tandem. **g** Schematic of the targeted transcriptional activation of porcine endogenous genes using the DIC system. **h**–**i** Q-PCR results in DIC PFFs transfected with MPH and different dgRNAs, presented as mean ± SEM, to measure the transcript level of *CDX2* locus (**h**) and *SOX2* locus (**i**) normalized to control cells from three independent experiments (*n* = 3). DIC PFFs transfected with only MPH were used as control cells

A total of 8 sgRNA-expressing vectors targeting porcine *TP53*, *APC*, *KRAS*, *OCT4*, *LMNA*, *ALK*, *EML4*, and *PCSK9* genes, respectively (Fig. 3a), were designed and transfected into DIC-F1-PFFs, which were then cultured in the medium supplemented with Dox. Sanger sequencing results of the PCR products corresponding to amplified targeted sites showed that induced SpCas9 could cut the porcine genome for all tested targeted sites (Additional file 1: Fig. S5). Quantitative assessment of Sanger sequencing results showed that the efficiency of genome editing ranged from 4.5% to 64.3% (Fig. 3b). Next, we further investigated whether the DIC system could be used to achieve large-scale chromosome genetic engineering, such as chromosome inversion and large fragment deletion (Fig. 3c). The tested *ALK*-sgRNA and/or *EML4*-sgRNA were transfected into DIC-F1-PFFs. PCR and Sanger sequencing results showed that the events of chromosome inversion and large fragment deletion were successfully induced in Dox-treated DIC cells with simultaneous delivery of *ALK*-sgRNA and *EML4*-sgRNA (Fig. 3d). RT-PCR and subsequent Sanger sequencing results of TA clones showed that the *EML4*-*ALK* fusion transcripts were also expressed in these cells (Fig. 3e). In addition, the cascade architecture of *ALK*-sgRNA linked *EML4*-sgRNA by pre-tRNA sequence could also result in chromosomal inversion and large fragment deletion in DIC-F1-PFFs with Dox treatment with comparable efficiency (Fig. 3d). These results confirmed that Dox-induced SpCas9 can engineer the porcine genome and generate the indels, chromosomal inversions, and large fragment deletions with specific sgRNAs.

### Targeted transcriptional activation of porcine endogenous genes by using the DIC system

Targeted gene activation is of critical importance for developing therapeutic targets and fundamental biology studies. However, endogenous target gene activation is difficult to implement. The CRISPR/Cas9-based system has been repurposed to enable enhanced gene expression or gene activation, which always relies on sgRNA to recruit dCas9 linked by transcriptional activation complexes to target loci [34]. To expand the applicability of the DIC system beyond genome editing, we next tested whether endogenous gene activation could be implemented through combination of dead sgRNA containing MS2-loop and transcriptional activation complexes [35, 36]. A cassette containing transcriptional activation complexes MS2-P65-HSF1 (MPH) and series engineered sgRNAs with additional MS2-loop was designed (Fig. 3f). To avoid the double stranded breaks created by the SpCas9 and sgRNA complex, modified sgRNAs carrying short target sequence (14-bp) were chosen to inactivate SpCas9 [35]. When Dox-treated DIC cells are transfected with plasmids expressing MPH activators and modified sgRNAs, the transcriptional machinery can be recruited to target loci by SpCas9 protein and MS2-looped sgRNA complex, and then activate or enhance the mRNA transcription, thereby increasing the expression levels of target genes (Fig. 3g). As proof-of-principle experiments, we designed and constructed six modified sgRNAs (dgRNA-1, dgRNA-2, dgRNA-3, dgRNA-4, dgRNA-5, and dgRNA-6) located within 100 bp upstream of the translational start site for the *CDX2* and *SOX2* loci, respectively (Fig. 3h, i). Six modified sgRNAs in tandem were also created to elicit stronger gene activation through previously reported golden gate assembly [37] (Fig. 3f). For the *CDX2* locus, the Q-PCR results suggested that all six p*CDX2*-dgRNAs can be used to increase the transcript level with 1.74- to 170.10-fold change. Up to 187-fold change was achieved using the six modified sgRNAs in tandem (Fig. 3h). For the *SOX2* locus, only p*SOX2*-dgRNA-4 and dgRNA-6 alone showed the ability to increase the gene expression with 3.8-fold change and 2.8-fold change, respectively. However, when the six dgRNAs were used in tandem, 15.1-fold change of *SOX2* expression was achieved (Fig. 3i).

### Development of a facile spatiotemporal gene knockout strategy based on the DIC system

**Fig. 4:**
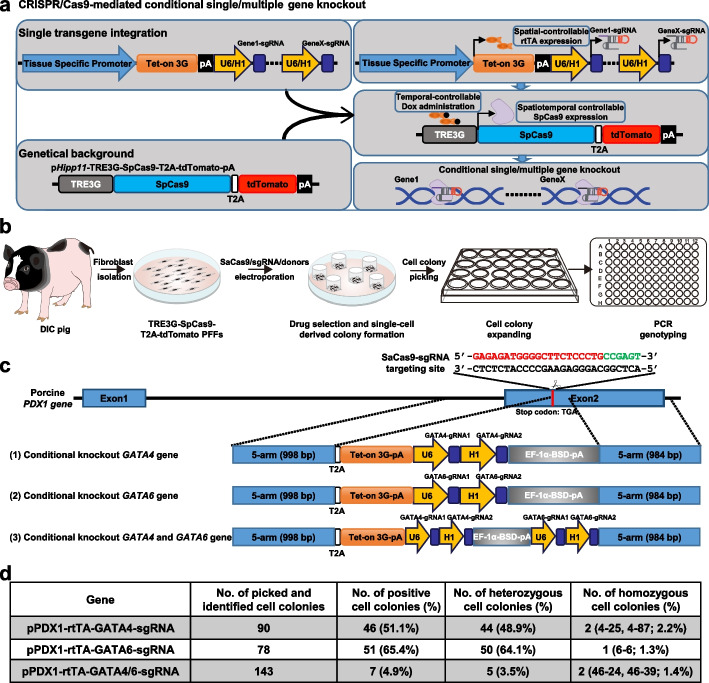
Simple strategy to achieve spatiotemporal control of one or more gene knockouts based on the DIC system. **a** Overview of the spatiotemporal control of one or more gene knockouts based on the DIC system. On the basis of PFFs carrying TRE3G-SpCas9-T2A-tdTomato cassette at the *Hipp11* locus, spatiotemporal gene knockout could be fulfilled only after transgene integration of spatial-specific-expressing rtTA and targeting interested-gene sgRNA expression cassette. **b** Outlined schematic for the establishment of cell line with spatiotemporal control of one or more gene knockouts based on the DIC system. **c** Schematic of the gene targeting strategy of *GATA4*, *GATA6*, and simultaneous *GATA4* and *GATA6* spatiotemporal gene knockouts. **d** Summary of cell screening experiments for *GATA4*, *GATA6*, and simultaneous *GATA4* and *GATA6* spatiotemporal gene knockouts

The DIC pig model allows flexible temporal control of SpCas9 activity in pigs via simple chemical induction at a specific time in vivo and in vitro but does not enable spatial-control of SpCas9 activity directly. Interestingly, on the basis of the DIC pig model, a construct containing 2A-linked rtTA-pA and one or multiple U6-sgRNAs cassette could be inserted into the downstream of tissue-specific expressing genes of PFFs carrying *Hipp11*-TRE3G-Cas9-T2A-tdTomato allele, which allows one-step generation of germline-inherited pigs enabling not only temporal but also spatial control of gene function in vivo under chemical induction (Fig. 4a, b). As a proof-of-principle experiment, the pancreas was selected as the first target organ to perform spatiotemporal gene knockout given that the uniformly high expression level of SpCas9 was observed in the pancreas derived from DIC pigs treated with Dox. Previous studies have found that GATA4 and GATA6 transcription factors in the pancreas play a key role in neonatal diabetes [38]. However, the function of GATA4 and GATA6 transcription factors in the pancreas remains elusive and appears different dosage sensitivity between humans and mice [38–40]. Therefore, we constructed three transgene cassettes to achieve spatiotemporal knockout of *GATA4* or *GATA6* or simultaneous knockout of *GATA4* and *GATA6* (Fig. 4c). We screened and selected sgRNAs for targeting the *PDX1*, *GATA4*, and *GATA6* loci in PFFs (Additional file 1: Fig. S6a-c). By using fibroblasts carrying TRE3G-driving SpCas9-T2A-tdTomato expression cassette at the p*Hipp11* locus, we picked 198, 268, and 257 single-cell derived colonies, respectively. For these colonies, 90, 78, and 143 were successfully expanded in 24-well plates and further screened by 5′- and 3′- junction fragment PCR. Genotyping PCR results showed that 46 (46/90, 51.1%), 51 (51/78, 65.4%) and 7 (7/143, 4.9%) colonies carrying transgene integration were successfully generated for *GATA4*, *GATA6*, and *GATA4* and *GATA6*, respectively (Fig. 4d; Additional file 1: Fig. S6d-f). When these cells were used as donor nuclei for SCNT, spatiotemporal knockout of *GATA4* or *GATA6* or simultaneous knockout of *GATA4* and *GATA6* pig lines can definitely be obtained through only one-step SCNT cloning in the future.

### Validation of the effectiveness of in vivo genome editing and generation of metastatic PDAC model by using DIC pigs

**Fig. 5:**
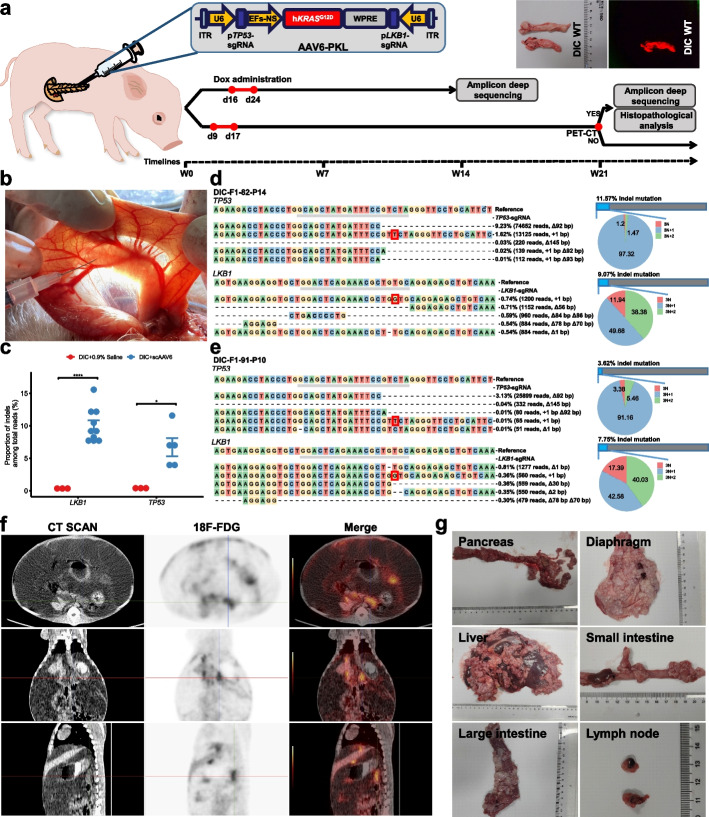
Effective in vivo genome editing and metastatic pancreatic adenocarcinoma modeling. **a** Schematic representation of the generation of pancreatic adenocarcinoma models with *TP53*^−/−^; *KRAS*^G12D^; *LKB1*^−/−^ (PKL). **b** Injection of AAV6-PKL or 0.9% saline into the main pancreatic duct or the body of the pancreas. **c** Indel frequency measured by amplicon deep sequencing of *TP53*-gRNA (*n* = 5), *P* = 0.0109, *P < 0.05 or *LKB1*-gRNA (*n* = 10), *P* = 9.13×10^−6^, ****P < 0.0001. Unpaired, two-tailed *t*-test was used for statistical analyses. **d**–**e** Classification of amplicon deep sequencing of pancreas tissue sections from DIC-F1-82-P14 (**d**) and DIC-F1-91-P10 (**e**) at p*LBK1* and p*TP53* loci, respectively. The representative top 5 indel reads were shown. **f** Post about 21-week AAV injection, PET-CT imaging for AAV6-PKL-injected DIC pig (DIC-F1-96). **g** Pictures of multiple organs from the abdominopelvic cavity of AAV6-PKL-injected DIC pig. Hyperplastic and adherent neoplasms were found in the liver, large intestine, diaphragm, and small intestine, and lymph nodes were enlarged

We next verified whether in vivo genome editing could be carried out by delivering sgRNA into DIC pigs accompanied with Dox induction. Given that the expression levels in the pancreas were relatively high after Dox induction, the pancreas was selected as the objective organ to model PDAC through mutating the tumor suppressor genes in vivo. AAV vectors containing sgRNAs targeting two tumor suppressor genes, p*TP53* and p*LKB*, as well as human *KRAS*^*G12D*^ expressing cassette, referred to as AAV-PKL, were constructed (Fig. 5a). Compared with AAV8 and AAV9, AAV6 could efficiently and safely target both normal and neoplastic pancreases via retrograde ductal delivery without inducing pancreatitis [41]. The AAV-PKL vectors were used for packaging AAV6-PKL. Four DIC-F1 piglets (DIC-F1-82, DIC-F1-86, DIC-F1-87, and DIC-F1-92; one for 55-day-old and three for 43-day-old) were injected with 2 × 10^12^ genome copies (GC) of AAV6-PKL into the main pancreatic duct and/or the body of the pancreas (Fig. 5b). One DIC-F1 piglet (DIC-F1-88) injected with equivalent volume of 0.9% saline was used as the control. The detailed information for injection and animals is summarized in Additional file 2: Table S1. Dox administration was performed at day 16 to day 24 post AAV-injection via oral administration combined with three intravenous injections every other day. To verify whether the predicted p*LKB1* and p*TP53* gene editing could be simultaneously achieved by AAV6-PKL delivered sgRNAs combined with Dox induction, these five piglets were sacrificed to harvest the whole pancreases after 14 weeks of AAV6 or saline injection. Each pancreas was then randomly dissected to 15 pieces (named as P1-P15 indicated in Additional file 1: Fig. S7a) and subjected to amplicon deep sequencing. The sequencing results showed that 11 (18.3%, 11/60) samples harbored gene editing at the p*TP53* and/or p*LKB1* loci, whereas gene editing was almost undetectable in DIC-F1 pigs injected with 0.9% saline. Of these 11 samples, 10 had indel mutations at the target site of p*TP53*-sgRNA with efficiencies ranging from 3.62% to 11.57%; 5 harbored indel mutations at the target site of p*LKB1*-sgRNA with efficiencies ranging from 7.41% to 15.56%; 4 simultaneously incorporated indel mutation at the p*TP53* and p*LKB1* loci (Fig. 5c; Additional file 1: Fig. S7a). We further analyzed the type and ratio of indel reads. The representative top 5 indel reads were listed in Fig. 5d, e and Additional file 1: Fig. S7b-j. We found that p*LKB1*-sgRNA and p*TP53*-sgRNA could induce more frequent out-of-frame mutation than in-fame mutation. More specifically, sgRNA targeting the p*LKB1* locus induced a large proportion of mutations with 3N + 1 or 3N + 2 bp insertions or deletions, whereas sgRNA targeting the p*TP53* locus induced predominant mutations with 3N + 1 bp insertions or deletions. These results suggested that Dox-induced SpCas9 can efficiently introduce target gene mutation with specific sgRNAs in vivo.

We further injected AAV6-PKL to three other DIC-F1 pigs with different dosages (3.5 × 10^12^ GC for DIC-F1-96, 3.0 × 10^12^ GC for DIC-F1-99, and 1.5 × 10^12^ GC for DICK-F1-1) (Additional file 2: Table S1) and raised the pigs for long term to determine whether pancreatic cancer could develop. Dox administration was performed at day 9 to day 17 post AAV injection via oral administration combined with three intravenous injections every other day (Fig. 5a). DIC-F1-99 pig died during Dox administration due to unknown reasons (Additional file 2: Table S1). The health status of the remaining two pigs was closely monitored by visual inspection and weight tracking. For DIC-F1-96, obvious abdominal distension and decreased ingestion were observed at about 21 weeks post AAV6 injection. Positron emission tomography-computed tomography (PET-CT) was performed for DIC-F1-96. The PET-CT results revealed that a huge mass in the abdominopelvic cavity and massive ascites; pancreatic metabolism was diffusely slightly high (Fig. 5f). DIC-F1-96 was then euthanized (Additional file 1: Fig. S8a). During autopsy, hyperplastic and adherent neoplasms in the liver, large intestine, diaphragm, and small intestine and enlarged lymph nodes were also found (Fig. 5g). These results suggested that pancreatic malignant tumor with multiple lymph node metastasis in the abdominal cavity possibly developed in DIC-F1 pig through in vivo genome editing.

**Fig. 6:**
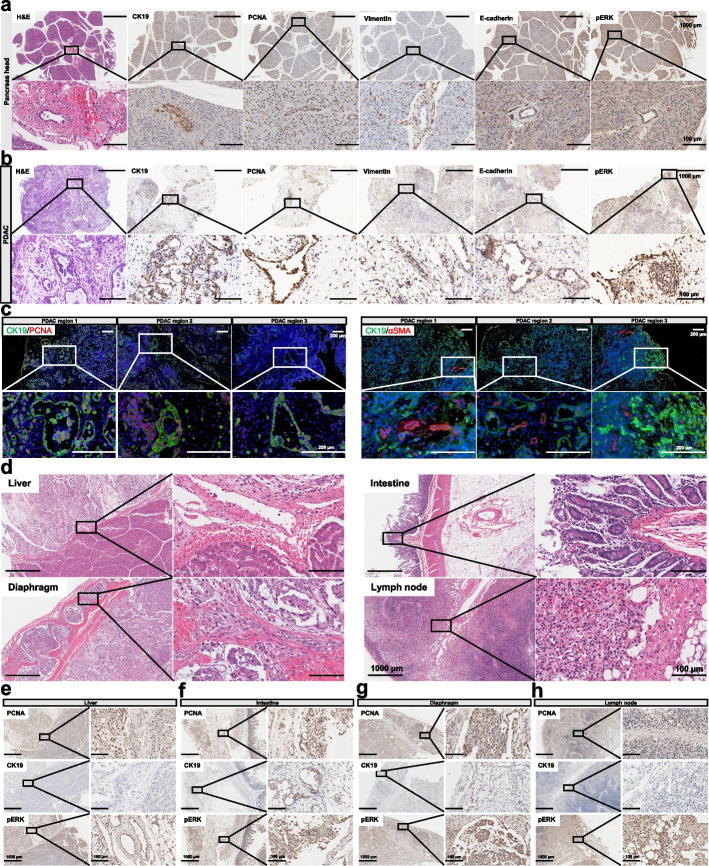
Histopathology of DIC-F1 pig with metastatic pancreatic adenocarcinoma. **a**–**b** H&E and IHC staining of sections from the different pancreatic head and tumor sites. CK19, a ductal origin marker. PCNA, a proliferative marker. Vimentin, a mesenchymal marker. E-cadherin, an epithelial lineage marker. pERK, a KRAS activation effector. Scale bars, 100μm. **c** Representative immunofluorescence staining results of different PDAC regions. CK19-positive cells were also PCNA positive. CK19 was positive at the membrane of tumor cells, whereas PCNA was positive at the nucleus of tumor cells. αSMA, pancreatic stellate cell marker. Scale bars, 200 μm. **d** H&E staining of other metastatic organs (liver, intestine, diaphragm, and lymph node) in PDAC pig. Scale bars, 1000 μm or 100 μm as indicated. **e**–**h** IHC staining sections from the liver (**e**), intestine (**f**), diaphragm (**g**), and lymph node (**h**) to analyze the expression of PCNA, CK19, and pERK, respectively. Scale bars, 1000 μm or 100 μm as indicated

**Fig. 7:**
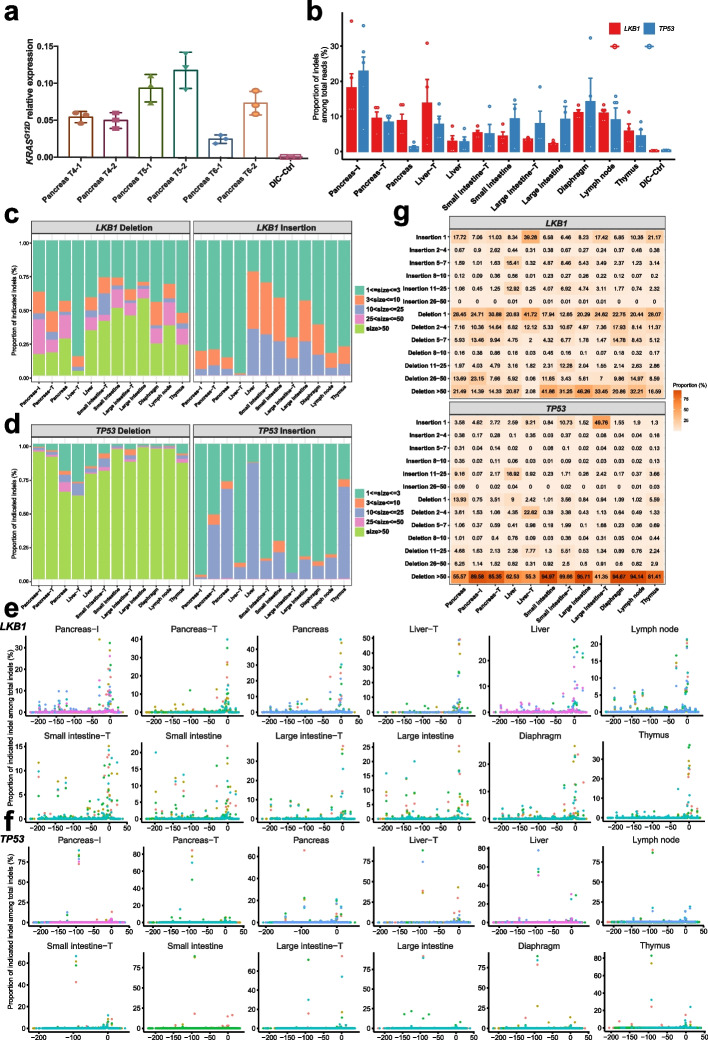
Analysis of oncogene *hKRAS*^*G12D*^ expression and tumor suppressor (*TP53*, *LKB1*) mutation in DIC pig with metastatic pancreatic adenocarcinoma. **a** Q-PCR analysis of h*KRAS*^*G12D*^ expression levels in six pancreatic tumor samples normalized to control samples from wild-type pigs. Means of three biological replicates were shown. Error bars indicate SEMs. **b** Indel frequency analysis by amplicon deep sequencing of *TP53*-gRNA or *LKB1*-gRNA targeting sites in the pancreas and other metastatic organs from DIC pig injected with AAV6-PKL. Data are plotted as mean ± SEM and each dot represents a different tissue section. **c**–**d** Mean proportions related to varied indel size for *LKB1*-gRNA targeting site (**c**) or *TP53*-gRNA targeting site (**d**) in the pancreas and other metastatic organs from DIC pig injected with AAV6-PKL. **e**–**f** Distribution of indel for *LKB1*-gRNA targeting site (**e**) or *TP53*-gRNA targeting site (**f**) in the pancreas and other metastatic organs from DIC pig injected with AAV6-PKL. Dots of one color represent one different tissue section. **g** Heatmap analysis for simultaneous length distribution and mean proportion of indel in *LKB1* and *TP53* loci, respectively, in the pancreas and other metastatic organs from DIC pig injected with AAV6-PKL

Given that we initiated porcine tumor formation through local injection of AAV6-PKL restricted to the main pancreatic duct to drive the transformation of ductal epithelial cells, we first evaluated whether PDAC was developed at the local injection site. Hematoxylin-eosin (H&E) staining results of the pancreatic head near the local injection site indicated that the pancreatic histological structure was disorganized, and nodular tumors occurred around the pancreatic duct (Fig. 6a). Upon histological staining of geographically separate regions, we found that porcine PDAC samples displayed typical lumenal masses with dominantly cellular atypia, which was consistent with the morphological features of human patients with PDAC. Porcine tumor sections were also extensively stained positive with Masson’s trichrome (Additional file 1: Fig. S8c), indicating that a desmoplastic tumor stroma developed similar to human patients with PDAC [42]. IHC staining of pancreatic head and tumor sections showed that pERK (a KRAS activation effector), PCNA (a proliferative marker), CK19 (a ductal origin marker), E-cadherin (an epithelial lineage marker), and vimentin (a mesenchymal marker) were expressed in the tumor stroma (Fig. 6a, b), indicating that PDAC had developed in the DIC pig. Dual immunofluorescence staining on PDAC samples showed that CK19-positive regions were also positive for proliferating marker, PCNA (Fig. 6c). Considering that pancreatic stellate cells are the critical mediator of tumor-associated fibrosis, we also assayed the expression of pancreatic stellate cell marker αSMA. The expression of αSMA was associated with areas of CK19-positive PDAC and exclusively localized to the tumor stroma (Fig. 6c). Similarly, pancreatic tumors from other separate areas also expressed E-cadherin, pERK, and vimentin in the tumor stroma (Additional file 1: Fig. S8b). Metastatic tumors in the liver, intestine, diaphragm, and enlarged lymph nodes were also sectioned and subjected to histological analysis. H&E staining results indicated that tumor cells were occasionally accompanied with invasion of inflammatory cells in the diaphragm, lymph node, liver and intestine (Fig. 6d). Severe tissue fibrosis occurred in neoplasms and adherent organs confirmed by Masson’s trichrome (Additional file 1: Fig. S8c). Consistent with primary pancreatic tumors, IHC staining was positive for PCNA and pERK in these metastases. CK19 expression could also be detected in metastatic tumor cells, but not in hepatocytes, lymphocytes or muscle cells (Fig 6e–h). Human *KRAS*^*G12D*^ expression was detected in all six analyzed pancreatic tumors through Q-PCR experiments with mutant KRAS specific primer pairs (Fig. 7a), but not in DIC control pig without AAV6-PKL injection.

To further evaluate the induced gene mutation at targeting sites, genomic DNAs were extracted from pancreatic head containing AAV6 injected sites (Pancreas-I), pancreatic tumors (Pancreas-T), metastatic tumors in liver (Liver-T), small intestine (Small intestine-T), large intestine (large intestine-T), tissues adjacent to tumors (Pancreas, Liver, Small intestine, Large intestine), and tissues from the diaphragm, thymus, and lymph node. We collected 3 to 6 samples of each group for statistical analysis. Next-generation sequencing (NGS) of PCR-amplified p*LKB1*-sgRNA and p*TP53*-sgRNA targeting sites using these genomic DNAs as template was then conducted. Indels at p*LKB1* and p*TP53* targeting sites were found in all tested tissues except that from un-induced DIC control pig (Fig. 7b). Indel frequency in tumors was obviously higher than that in the pancreas and liver adjacent to tumors. The highest indel frequency reached 37.1% for p*LKB1* and 35.8% for p*TP53* in Pancreas-I. Interestingly, comparable indel frequency in tumors from the small/large intestine and in adjacent tissues was found, indicating a high ratio invasion of metastatic tumor cells into intestines. The thymus and lymph node with detectable indel could be another direct evidence of malignant tumors accompanied with lymph node metastasis (Fig. 7b). By statistically analyzing indel reads, small deletion (1–3 bp), large deletion (> 50 bp), and small insertion (1–3 bp) were the dominant mutation types for the *LKB1*-sgRNA targeting site (Fig. 7c), whereas large deletion (> 50 bp) and small insertion (1–3 bp) were the dominant mutation types for the p*TP53* targeting site (Fig. 7d). Although the indel sizes and positions varied between each group, we detected the most variable mutation types in pancreatic tissues, and the most frequent mutant DNA base pair covered the predicted SpCas9 cleavage sites, both for the p*LKB1* and p*TP53* loci (Fig. 7e, f). Combining statistical heatmap and mutation type analysis, several indel reads incorporating specific mutation types, such as insertion/deletion 1 bp mutation at p*LKB1* targeting site and deletion 92 bp at p*TP53* targeting site, were enriched during tumor formation and malignancy (Fig. 7e–g). Of particular note is the specific mutation type of deletion 92 bp at p*TP53* targeting site, which had predominant enrichment among varied indel reads in most of detected tumor and adjacent tissues (Fig. 7e–g). These enriched indel reads possibly reflect specific mutation types which can empower tumor cells with survival, proliferative, and metastatic advantages.

## Discussion

In vivo genetic perturbation strategy has been developed with the advent of powerful CRISPR-Cas9-based technologies [12–14]. However, the large size of SpCas9 (~4.2kb) is unsuitable for effective in vivo delivery. SpCas9-expressing mouse models have been generated to overcome this hurdle [16, 43, 44]. For pigs, transgenic models with ubiquitously constitutive Cas9 expression [19, 45] and Cre-inducible Cas9 expression [25] have been reported previously. Here, we generated and validated the application of a transgenic pig line with Dox-inducible Cas9-expression through precise knock-in of the binary Tet-On elements into the *Rosa26* and *Hipp11* loci, respectively. The site-directed transgene integration could assure a more controllable and uniform expression level of SpCas9 among different individual pigs and derived cells and is favorable for germline transmission in offspring. With this pig model, conditional in vivo and/or in vitro gene editing, chromosome engineering, and regulation of endogenous target gene for gain of function could be realized.

Compared with the transgenic pig line with ubiquitously constitutive Cas9 expression, DIC pig line has several advantages. First, inducible Cas9 expression system with flexible SpCas9 switch is able to limit SpCas9 expression in a desirable time window, thus reducing the potential toxic effects of sustained SpCas9 expression, including DNA damage [20, 21, 46], chromosome instability [47], immune response [24], embryo lethality [19], p53 mutant enrichment, and potential carcinogenicity [20, 46, 48]. Second, inducible SpCas9 expression enables decreasing off-target events through limiting the duration of SpCas9 presence after on-target cleavage. Off-targets could confound the phenotypes of GE animal models [49]. Third, inducible Cas9 expression system can be employed to carry out studies such as cell lineage tracing, which requires tight regulation of SpCas9 expression time and dosages [50]. Compared with the present chemical inducible SpCas9 pigs, the widespread applications of the Cre-inducible SpCas9-expressing pig line [25] previously generated by our group are limited by several factors. First, unlike the mouse model, very few established genetically engineered Cre-expressing pig models required for intercrossing with Cre-inducible SpCas9-expressing pig are available because the generation is expensive and time-consuming. Second, two sequential recombination events mediated by Cre-recombinase compromise the activation of SpCas9 in specific tissues or organs, thus decreasing the genome editing scope and efficacy. Third, the expression of latent SpCas9 is sustained after a single activation by Cre-recombinase, which cannot avoid potential toxic effects as those of constitutive SpCas9 expression. DIC pigs could overcome the shortcomings above. Furthermore, in contrast to complicated viral delivery of Cre into utero, we confirmed that Dox could overcome the porcine placental barrier and induce high level of SpCas9 expression in the fetuses within utero after Dox treatment of pregnant sows. Moreover, the inducer (Dox) is cheaper and easier to deliver to target cells/tissues in vivo than Cre-recombinase.

Traditional and widespread methods to achieve spatiotemporal control of gene knockout require the creation of a DNA sequence flanked by a pair of *lox*P (floxed) sites through precise knock-in of the floxed donor template and deletion of a floxed DNA sequence followed by Cre-mediated recombination reaction [28]. Simultaneous multiple conditionally gene knockout, requiring multiple knock-in at different gene loci, greatly increases the complexity. Here, on the basis of the DIC system, we described a facile one-step GE strategy, in which a single knock-in of transgene cassette carrying sgRNA and rtTA driven by a tissue-restricted promoter, enabling simultaneous Dox-dependent temporal specificity and tissue specificity for SpCas9 regulation. Thus, spatiotemporal control of gene knockout is achieved. Conditional multiple gene knockouts can be achieved through merely adding more sgRNAs targeting different genes to the single transgene cassette. Moreover, cell types, tissues, or organs that are difficult to transduce with viruses carrying sgRNAs can also be amenable to conditional gene editing using this strategy.

Our group has long been interested in generating primary porcine cancer models [25]. The antitumor systems of animals are thought to co-evolve with their body size and longevity [51]. Compared with small animals, larger long-lived mammals actually get similar or less frequency of tumorigenesis, which indicates that larger animals have evolved more similar mechanisms to humans in cancer biology [52]. Moreover, given the more human-like size, anatomy, physiology, immune system, and life expectancy compared with rodents, porcine cancer models may allow the development of interventional surgical, radiological, and immunological therapy strategies that are more applicable to human patients. Pancreatic cancer is one of the most fatal and aggressive malignancies with poor prognosis. Up to 85% of patients with PDAC always present with either locally advanced or distant metastatic tumors [53]. Therefore, suitable animal models mimicking PDAC patients are urgently required for exploring new early diagnosis and effective therapeutic strategies. Here, by using the DIC system, we validated effective gene targeting with tumor suppressor genes such as *TP53*, *APC*, and *LKB1*; oncogene *KRAS*; and tumor-driven fusion genes such as *ALK* and *EML4* in vitro. Through the AAV6 delivery of sgRNAs into DIC pigs, we validated effective gene targeting with tumor suppressors *TP53* and *LKB1* in adult pig pancreas. Furthermore, we established the porcine PDAC model with consistent histopathological features with PDAC patients. Combining analysis of statistical results of amplicon deep sequencing, we identified that the mutation type of 92 bp deletion at the p*TP53* targeting site has a predominant enrichment in most of detected tumor tissues or adjacent tissues, indicative of powerful genetic mutation driver of tumor formation or metastases.

## Conclusions

In summary, the porcine DIC system established in this study provides a facile and tunable genetic engineering basis. With this pig model, genetic perturbation of gene loci, including loss-of-function and gain-of-function, can readily be performed by a single delivery of engineered sgRNAs or combined with transcript activators. Moreover, this DIC pig line can also be used as a basic tool to develop a single transgene integration strategy that allows the generation of spatiotemporal gene knockout lines, empowering the preclinical replication of complicated diseases and more refined gene function dissection. DIC pigs with a flexibly controlled SpCas9 expression are particularly indispensable for studies highlighting the SpCas9-expressing dosage and time window, such as toxic effect of SpCas9 protein and linage tracing studies. In a proof-of-concept trial for validation of the application, we achieved effective in vivo gene editing and thus rapidly established a metastatic PDAC pig model mediated by tumor suppressor *TP53* and *LKB1* inactivation and *KRAS*^*G12D*^ expression.

## Materials and methods

### Plasmids

Targeting plasmid pFlexible-Hipp11-TRE3G-tdTomato and AsCpf1 expressing plasmid were described in our previous study [31]. To construct targeting vector, pFlexible-Hipp11-TRE3G-SpCas9-T2A-tdTomato, SpCas9-T2A-tdTomato fragment was PCR-amplified from pFlexibleDT-pRosa26-iCas9 targeting plasmid reported previously [29] and subsequently assembled with Nhe I (Thermo Fisher) and Pme I (Thermo Fisher) digested pFlexible-Hipp11-TRE3G-tdTomato targeting plasmid using ClonExpress MultiS One Step Cloning Kit (Vazyme). The sgRNAs targeting porcine endogenous genes were designed and cloned into BbsI-digested U6-gRNA-expressing plasmids (Addgene 48962) or H1-gRNA-expressing plasmids (Addgene 53186) by primer pair annealing and T4 DNA ligase (NEB).

Short dgRNAs for gene activation were selected within − 150 and + 150 bp of the translation initiation site (ATG) and cloned into BbsI-digested sgRNA (MS2)_cloning_backbone (Addgene 61424). MPH transactivation domain from the plasmid lenti_MS2-P65-HSF1_Hygro (Addgene 61426) was cloned into the downstream of the CMV promoter. Epigenomic editing constructs harboring six sgRNA blocks, driven by the U6 promoter, were assembled by the Golden Gate assembly method [35]. Briefly, the single dgRNA expressing cassette was PCR-amplified using specific primers with overhangs and assembled together by T4 DNA ligase (NEB) and BpiI (Thermo Fisher) with the cycling parameters of 10 cycles at 37 °C for 5 min, 16 °C for 5 min, and 80 °C incubation for 20 min.

For spatiotemporal gene knockout, targeting vector, p*PDX1*-rtTA-*GATA4*-sgRNA, p*PDX1*-rtTA-*GATA6*-sgRNA, and p*PDX1*-rtTA-*GATA4/6*-sgRNA, were harvested by orderly assembling of diverse PCR fragments containing T2A-rtTA, drug selection cassette, sgRNA expressing cassette, 988 bp 5′homology arm, and 984 bp 3′ homology arm into Pme I and Not I-digested pFlexibleDT vectors using pEASY-Uni Seamless Cloning and Assembly Kit (TransGen).

All primers were synthesized by GENEWIZ, and all plasmids were validated via Sanger sequencing by Guangzhou IGE Biotechnology. All primer sequences are provided in Additional file 3: Table S2.

### Establishment of DIC and Cas9-based condition knockout fibroblasts, and DIC pig lines

The pFlexible-Hipp11-TRE3G-SpCas9-T2A-tdTomato vectors (10 μg), AsCpf1 (10 μg), and Hipp11-Cpf1-sgRNA (3 μg) expressing vectors were co-electroporated into pRosa26-rtTA PFFs under the condition of 1350 V, 30 ms, and 1 pulse using the Neon Transfection System (Life Technology). All primary porcine fibroblasts were cultured in high-glucose DMEM (HyClone) supplemented with 15% fetal bovine serum (Gibco), 1× Non-Essential Amino Acids (Gibco), 1× GlutaMax (Gibco), 1× sodium pyruvate (Gibco), and 1× penicillin-streptomycin (Gibco) and maintained in a cell incubator at 38.5 °C and 5% CO_2_. After 24-h recovery, the electroporated PFFs were equally divided into twenty 10-cm culture dishes with 5000 cells each. Puromycin (400 ng/mL) was added to select positive cells. After about 10 days, single-cell derived colonies were picked. After lysing a small fraction of the selected cell colonies with 10 μL of lysis buffer (0.45% NP-40 plus 0.6% Proteinase K) for 90 min at 56 °C and 15 min at 96 °C, lysates were harvested and analyzed by PCR using the Hipp11 genotyping primers. Positive cell colonies with correct gene knock-in were expanded and cryopreserved for SCNT. Screened DIC PFFs were thawed before SCNT. The SCNT protocol was conducted as described in our previous studies [4, 7–9, 25], and the surrogate sows delivered the cloned piglets naturally. Porcine ear fibroblasts (PEFs) derived from the cloned DIC (F0) founders were also isolated and cryopreserved. The F1 KI pigs were generated by crossing the male DIC (F0) founders to WT female Bama or Tibetan miniature pigs. Cell lines for spatiotemporal gene knockout were generated by electroporation, drug screening (blasticidin selection, 3 μg/mL), and PCR genotyping similar to the above description.

### PCR genotyping

Genomic DNAs were extracted from the ear tissues of newborn piglets (DIC Founders and F1) using TIANamp Genomic DNA Kit (TIANGEN) according to the manufacturer’s instructions. Then genomic DNAs were then used as a PCR template for the identification of gene knock-in at the *Rosa26* and *Hipp11* loci (primer pairs listed in Additional file 3: Table S2).

### Dox treatment

Dox delivery methods and concentrations were adapted from our previous study [31]. Unless indicated otherwise, Dox was administered to pigs via drinking water for 1 week (2 mg/mL mixed with 10 mg/mL of sucrose) and three intraperitoneal injections (50 mg/kg) every other day. For interrogating Dox-inducible Cas9 expression in porcine fetuses, Dox was administered to pregnant females via feeding for one week (50 mg/kg every day) and three intravenous injections (50 mg/kg) every other day. For all experiments, cultured DIC fibroblast cells or re-constructed DIC embryos by SCNT were treated with 10 μg/mL of Dox. Small-molecule AZA (Sigma) at a concentration of 50 μM was also added to the culture medium to enhance the expression of Dox-inducible systems in vitro.

### RT-PCR and Q-PCR

Total RNAs from cells or tissues were extracted using TRIzol (Invitrogen). We performed reverse transcription reactions by using approximately 500 ng of RNA as template in a 10-μL reaction volume with the PrimeScript RT Reagent Kit with gDNA Eraser (Takara) following the manufacturer’s protocol. Using the cDNAs as template, RT-PCR was performed by using 2× Rapid Taq Master Mix (Vazyme) to detect the EML4-ALK fusion transcripts. To assay the gene activation or induced SpCas9 expression, Q-PCR was also conducted using the TB Green PCR Master Mix (Takara) in Bio-Rad CFX96 in a 20-μL reaction volume according to the recommended protocol. Data were analyzed using the 2-ΔCt method. The primers used in RT-PCR or Q-PCR are listed in Additional file 3: Table S2.

### Flow cytometry analysis

For analyzing the percentage of tdTomato expression in porcine PBMCs, whole blood samples were subjected to erythrocyte lysis using the ACK Lysis Buffer (Beyotime), and washed and resuspended with 1× PBS. For PEFs or PFFs, cell samples were prepared by using 0.25% trypsin to dissociate adherent cells, and then washed and resuspended with 1× PBS. The high-quality samples were finally analyzed by using an Accuri C6 flow cytometer (Accuri Cytometers).

### Western blotting

Tissues were collected from different organs of DIC pigs treated or untreated with Dox and stored at − 80 °C. Approximately 20–50 mg of tissue was grinded to extract total protein using RIPA (Sigma), PMSF, and protease inhibitor (Thermo fisher). BCA Protein quantification kit (GenStar) was used to assay the concentration. Extracted total protein was boiled at 100 °C for 15 min. Then, these samples were subjected to sodium dodecyl sulfate-polyacrylamide gel electrophoresis (SDS-PAGE) and transferred onto PVDF membrane (Millipore). Next, 5% BSA (Sigma) was used to block the PVDF membrane for 2–3 h and incubated with diluted Cas9 antibody (1:2000) at 4 °C overnight. After washing three times with TBST buffer, the PVDF membrane was incubated with diluted HRP-Goat Anti-Rabbit IgG (1:5000) at room temperature for 2 h, and then washed with TBST three times. Finally, the 1:1 freshly mixed ECL hypersensitive luminescence solution A and B was added to the PVDF membrane to detect the protein band using MiniChemi^TM^830.

### Immunohistochemical and immunofluorescence staining

For immunohistochemical staining, fresh tissue samples from dissected pigs were collected and soaked in 4% paraformaldehyde at room temperature for 24–48 h, then embedded in paraffin, and finally sectioned. Dried slices were dehydrated with xylene and different concentrations of ethanol. Antigen recovery was conducted in citrate buffer at 100 °C for 10 min and cooled to room temperature. Next, 3% hydrogen peroxide was dropped in the section to block peroxidase. After washing with PBS, slices were blocked with goat serum for 1 h. Then, they were incubated with goat anti-Cas9 antibody overnight at 4 °C in a wet box and then incubated with HRP-conjugated rabbit anti-goat IgG antibody on the second day. Finally, the slices were treated with DAB and sealed with neutral gum for observation. For immunofluorescence staining, fresh tissue samples were embedded by OCT (SAKURA) and sectioned with a freezing microtome (Leica). Slices were dehydrated and fixed with cold acetone for 15 min. Slices were permeabilized with 0.5% Triton X-100 (Sigma) for 15 min and blocked with 10% goat serum for 2 h. Next, slices were incubated with Cas9 antibody (HuaBio) overnight at 4 °C. On the second day, the slices were washed with PBS and incubated with secondary antibodies (Proteintech) for 2 h at room temperature. After washing with PBS three times, sealing agent containing DAPI was used to seal the slices. These slices were finally observed under a confocal microscope (Leica).

### Animal surgical procedure for in vivo delivery of AAV6 and PET-CT

Detailed information about pigs and AAV6 dosage used for surgical procedure is provided in Additional file 2: Table S1. Pigs were fasted 24 h before surgery. For all cases, anesthesia was induced with Telazol, Ketamine, and Xylazine. Pigs were intubated in dorsal recumbency and maintained in a surgical plane of anesthesia with isoflurane. The pancreas was accessed surgically through right ventral paramedian incision and injected with AAV6-PKL. Pancreatic ducts enter the duodenum in the proximal portion in the right upper quadrant. For euthanasia, each animal was sedated and euthanized with sodium pentobarbital overdose. For in vivo imaging, animals were sedated as described previously and the imaging agent 18F-FDG (Gosun Cyclotron Medicine) was delivered intravenously, allowing for visualization of potential pathologies of whole body by PET-CT.

### Sanger sequencing and indel detection

Dox-treated DIC PFFs transfected with sgRNAs were collected and lysed with NP40 to perform PCR using each primer pairs (Additional file 3: Table S2) 2 or 3 days after transfection. As for tissue samples, we extracted genomic DNAs with TIANamp Genomic DNA Kit (TIANGEN) to be used as PCR templates. All PCR reactions were conducted by using 2×Rapid Taq (Vazyme) under the recommended procedure. PCR products with correct size were purified by HiPure Gel Pure DNA Mini Kit (Magen) and subjected to Sanger sequencing. Finally, the results of Sanger sequencing were analyzed by a Web tool (https://tide.nki.nl) [54].

### Deep sequencing and analysis

To dissect the more detailed gene editing events around the genomic regions targeted by the sgRNA, we used custom amplification primers containing Illumina forward and reverse adapters to prepare the PCR library for NGS. First, we conducted 10-μL PCR reaction using 2× Phanta (Vazyme) to amplify the gRNA targeted regions. Subsequently, we took 50-μL PCR reaction containing 2 μL products from the first step, 25 μL 2× Phanta (Vazyme), and Illumina sequencing adapters. Purified and qualified PCR libraries were sent to Annoroad Gene Technology Corporation (Beijing) for NGS. The NGS data were analyzed by CRISPResso2 [55] and customed R scripts.

### Statistical analysis

Independent replicate experiments were conducted for statistical analysis. Three technical replicates with at least three biological sample replicates were included for Q-PCR experiments. The statistical analysis of Q-PCR results was conducted by using Prism 7 (GraphPad), and the results were displayed as mean ± SEM. As for amplicon deep sequencing data, more than three replicates were included, and statistical results were presented as mean ± SEM by using R packages. *P*-values were calculated by using unpaired, two-tailed *t*-test, and *P* value < 0.05 was considered as statistically significant.

## Supplementary Information

Additional file 1. Figures S1-S8 with figure legends for Doxycycline-dependent Cas9-expressing pig resources for conditional in vivo gene nullification and activation.

Additional file 2. Table S1 for Doxycycline-dependent Cas9-expressing pig resources for conditional in vivo gene nullification and activation.

Additional file 3. Table S2 for Doxycycline-dependent Cas9-expressing pig resources for conditional in vivo gene nullification and activation.

Additional file 4. Review history.

## Data Availability

All data supporting the findings in this study are available within this Article and its additional files. The raw data of deep sequencing are available at Genome Sequence Archive (https://bigd.big.ac.cn/gsa/browse/CRA006896) [56] or NCBI database (https://www.ncbi.nlm.nih.gov/bioproject/PRJNA812347) [57]. All other source data generated during this study are available from the corresponding authors upon reasonable request.
