# Supplementary material for: Doxycycline-dependent Cas9-expressing pig resources for conditional in vivo gene nullification and activation
Source: Genome Biol. 2023 Jan 17;24:8. doi: 10.1186/s13059-023-02851-x (PMC9843877; doi:10.1186/s13059-023-02851-x)
Supplement: Supplementary file 1 — Additional file 1. Figures S1-S8 with figure legends for Doxycycline-dependent Cas9-expressing pig resources for conditional in vivo gene nullification and activation. [file 13059_2023_2851_MOESM1_ESM.docx]

**
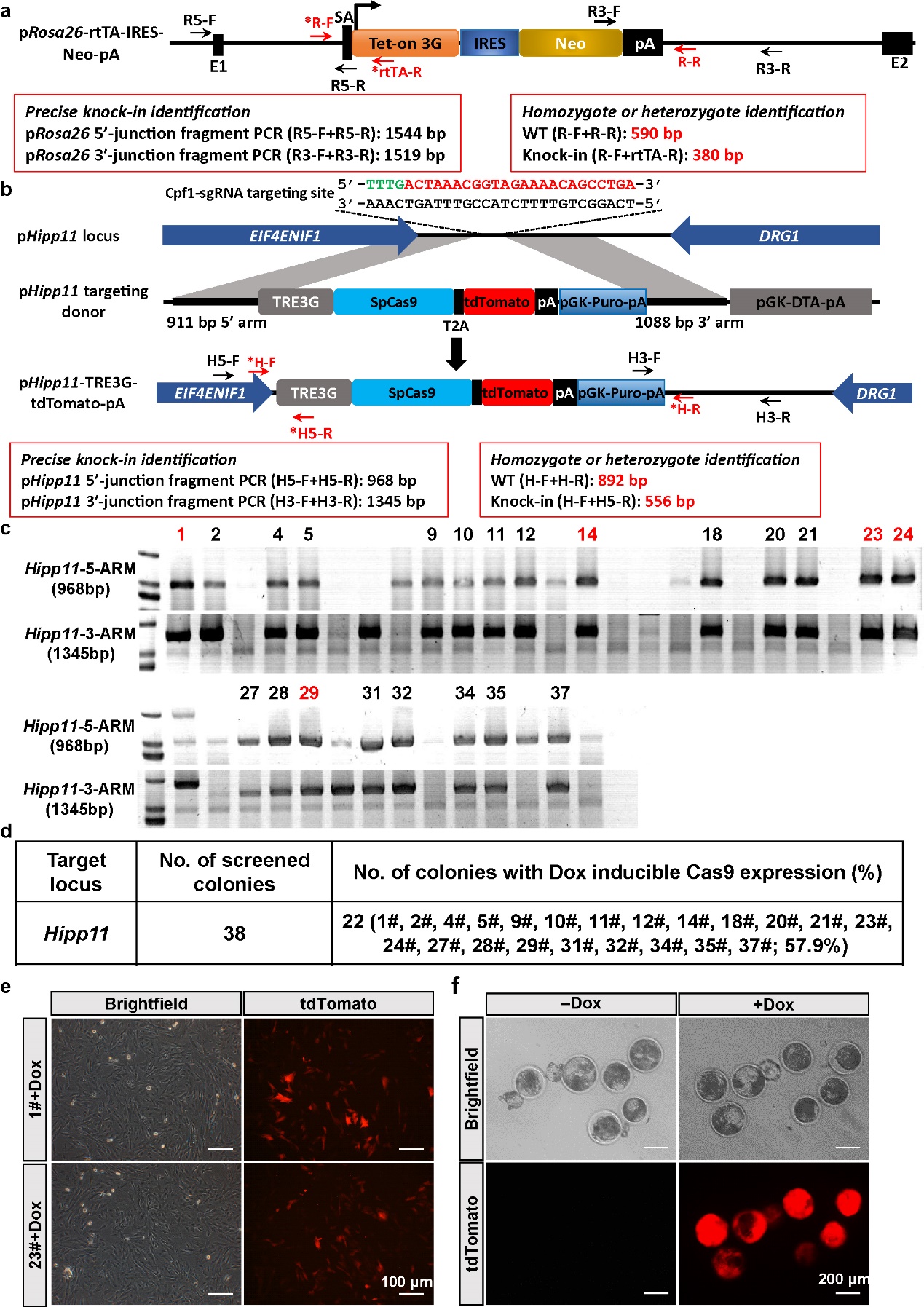
**

**Fig. S1. Generation and characterization of DIC PFFs for further SCNT.**

**a** Scheme for the rtTA-expressing cassette inserted into the p*Rosa26* locus. **b** Scheme for the integration of TRE3G-driving SpCas9-T2A-tdTomato-expressing cassette into the p*Hipp11* locus via CRISPR/Cpf1-mediated HDR. In **Fig. S1a** and **b**, PCR primers used for knock-in identification and homozygote or heterozygote identification were indciated by black and red arrowheads, respectively. **c** PCR genotyping results to analyze the precise knock-in events at the p*Hipp11* locus. **d** Statistics of detailed PFF colonies with TRE3G-SpCas9-T2A-tdTomato-expressing cassette knocked into the p*Hipp11* locus. **e-f** Fluorescent images of DIC colony 1# and 23# or reconstructed embryos by using DIC cells as SCNT-donor nuclei treated or untreated with Dox.

**
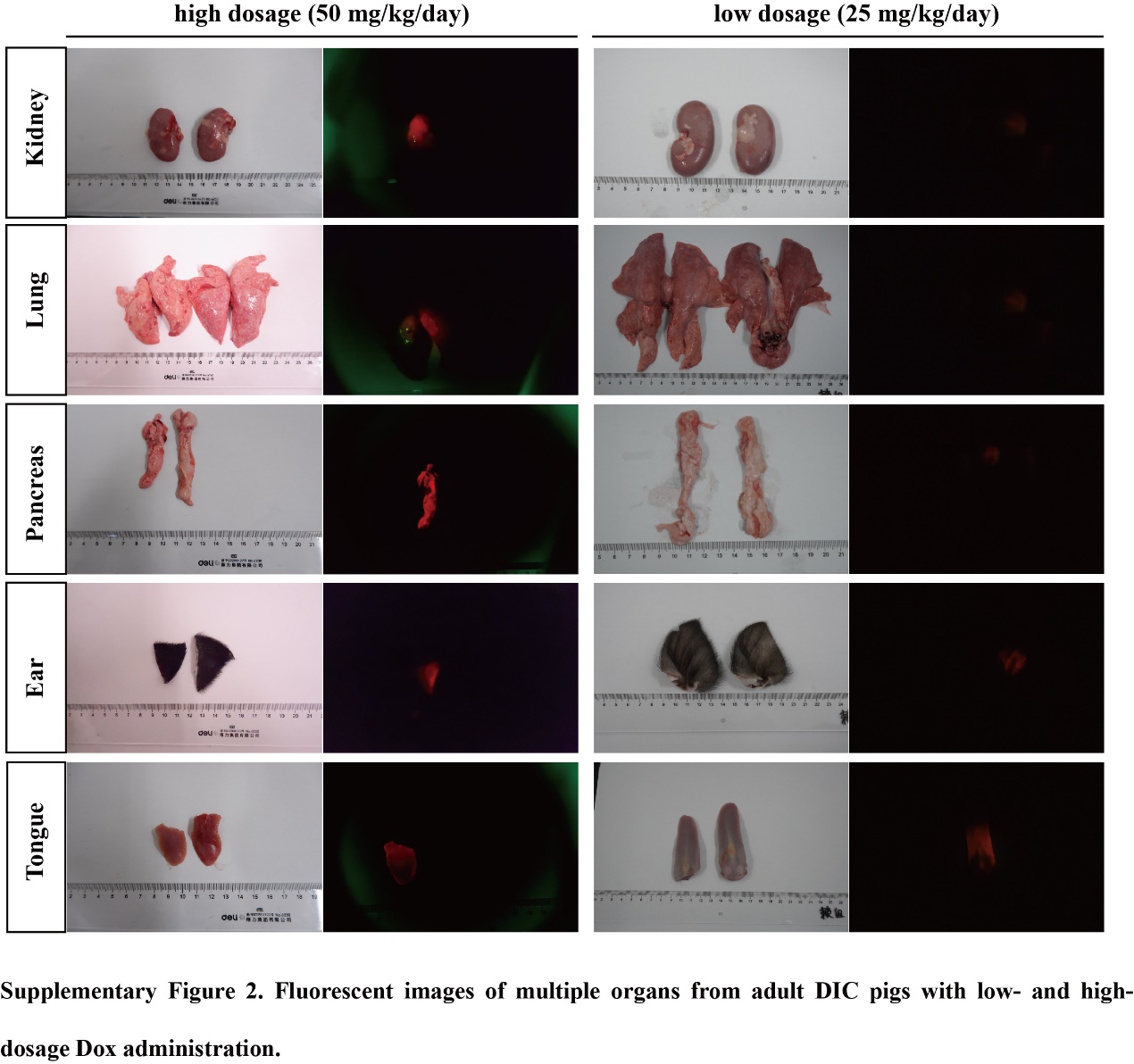
**

**Fig. S2. Fluorescent images of multiple organs from adult DIC pigs with low- and high-dosage Dox administration.**


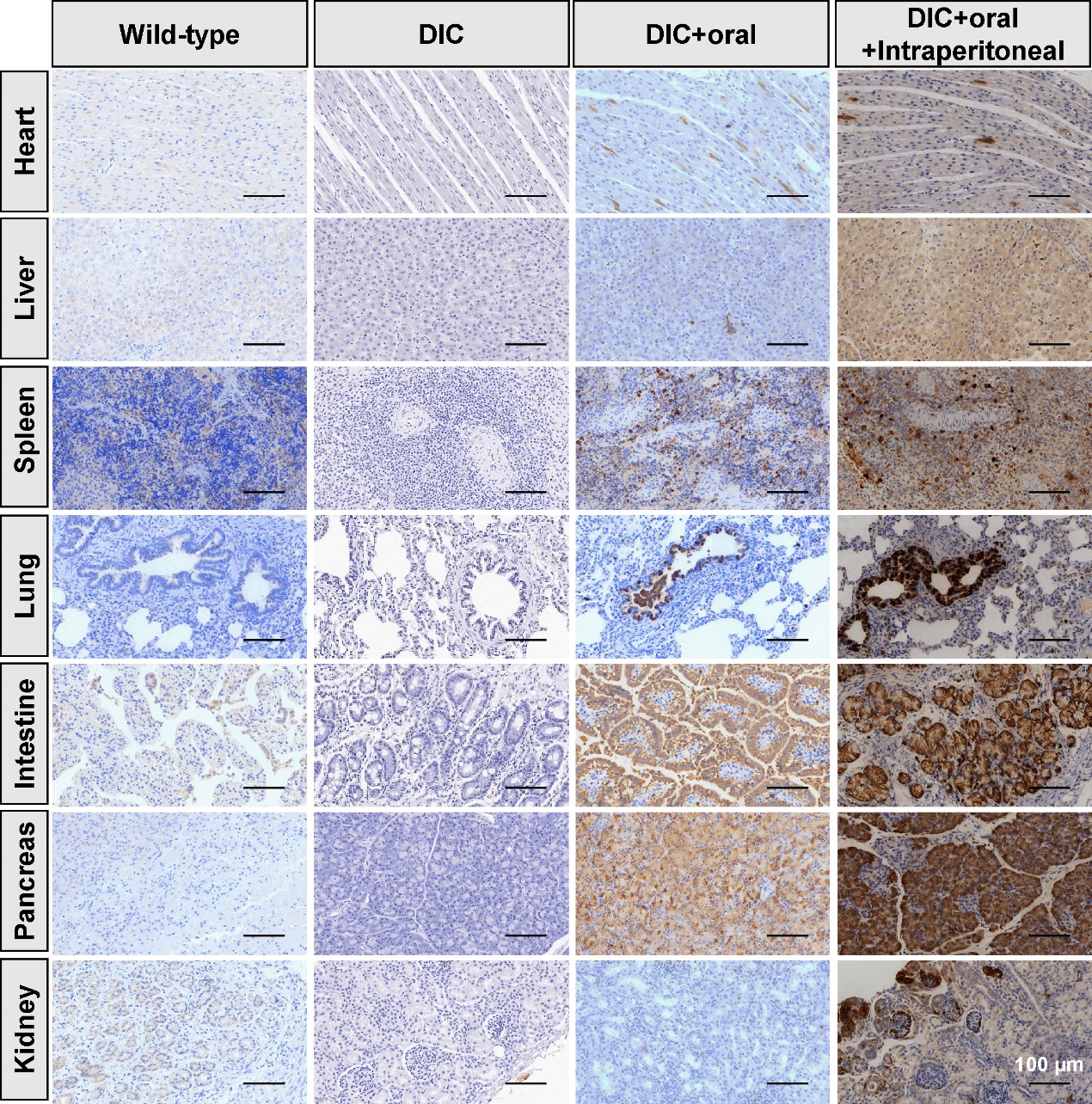


**Fig. S3. IHC staining results for comparison of SpCas9 expression induced by different Dox administration methods in multiple organs from adult DIC pigs. Scale bars, 100 μm.**

**
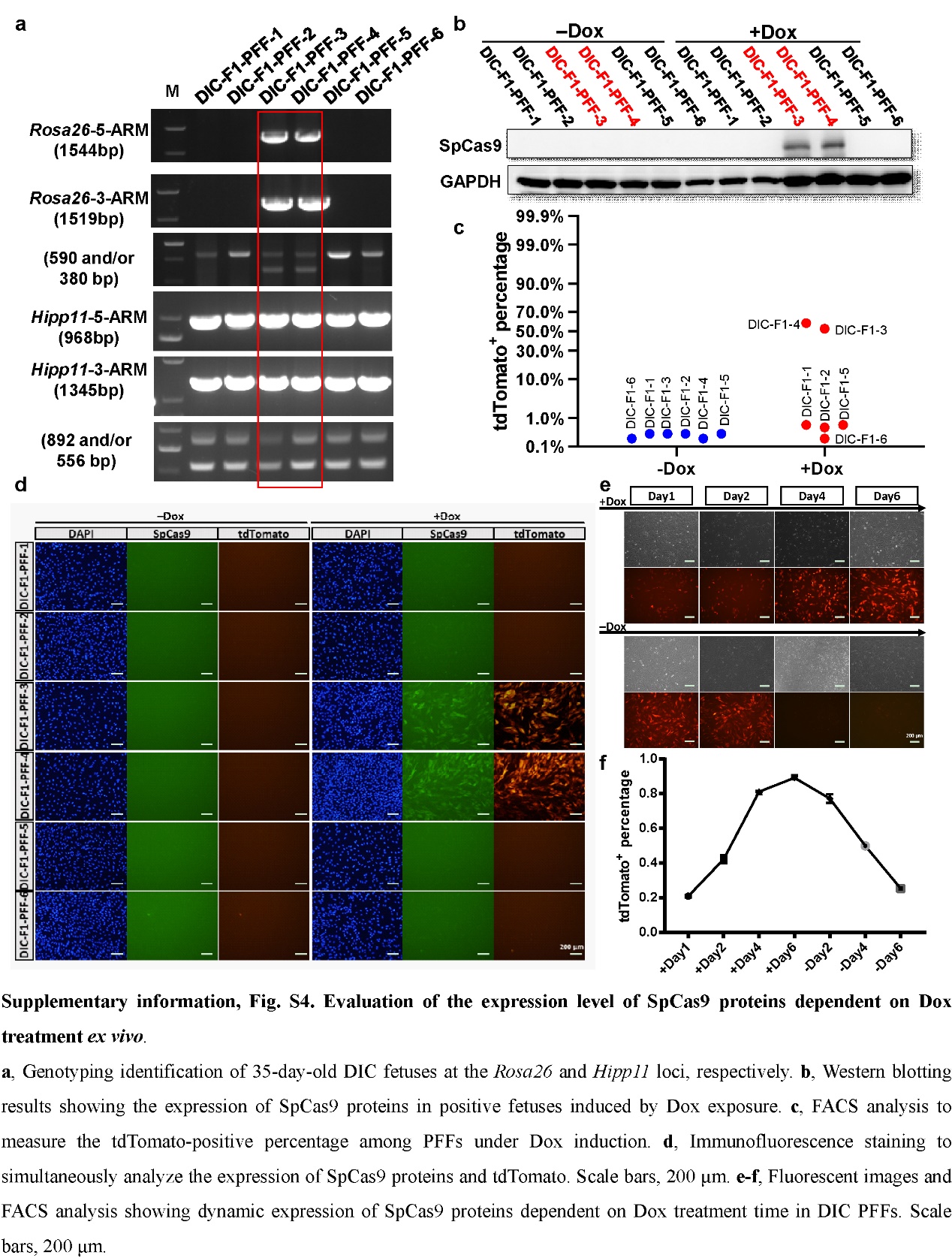
**

**Fig. S4. Evaluation of the expression level of SpCas9 proteins dependent on Dox treatment *ex vivo***.

**a** Genotyping identification of 35-day-old DIC fetuses at the *Rosa26* and *Hipp11* loci, respectively. **b** Western blotting results showing the expression of SpCas9 proteins in positive fetuses induced by Dox exposure. **c** FACS analysis to measure the tdTomato-positive percentage among PFFs under Dox induction. **d** Immunofluorescence staining to simultaneously analyze the expression of SpCas9 proteins and tdTomato. Scale bars, 200 μm. **e**-**f** Fluorescent images and FACS analysis showing dynamic expression of SpCas9 proteins dependent on Dox treatment time in DIC PFFs. Scale bars, 200 μm.

**
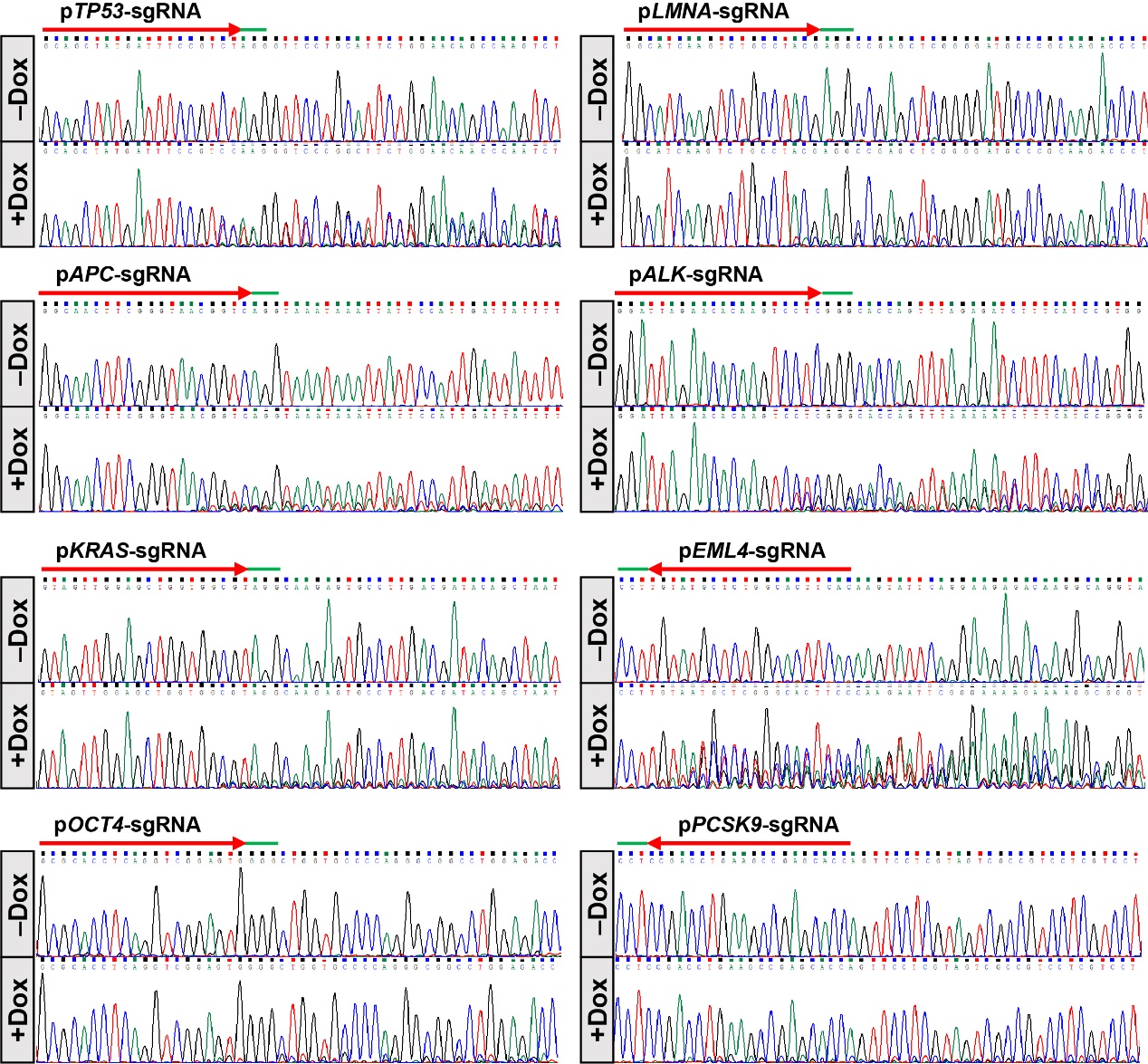
**

**Fig. S5. Sanger sequencing results showing effective gene editing achieved on the basis of Dox-treated DIC PFFs at multiple sites.**

**
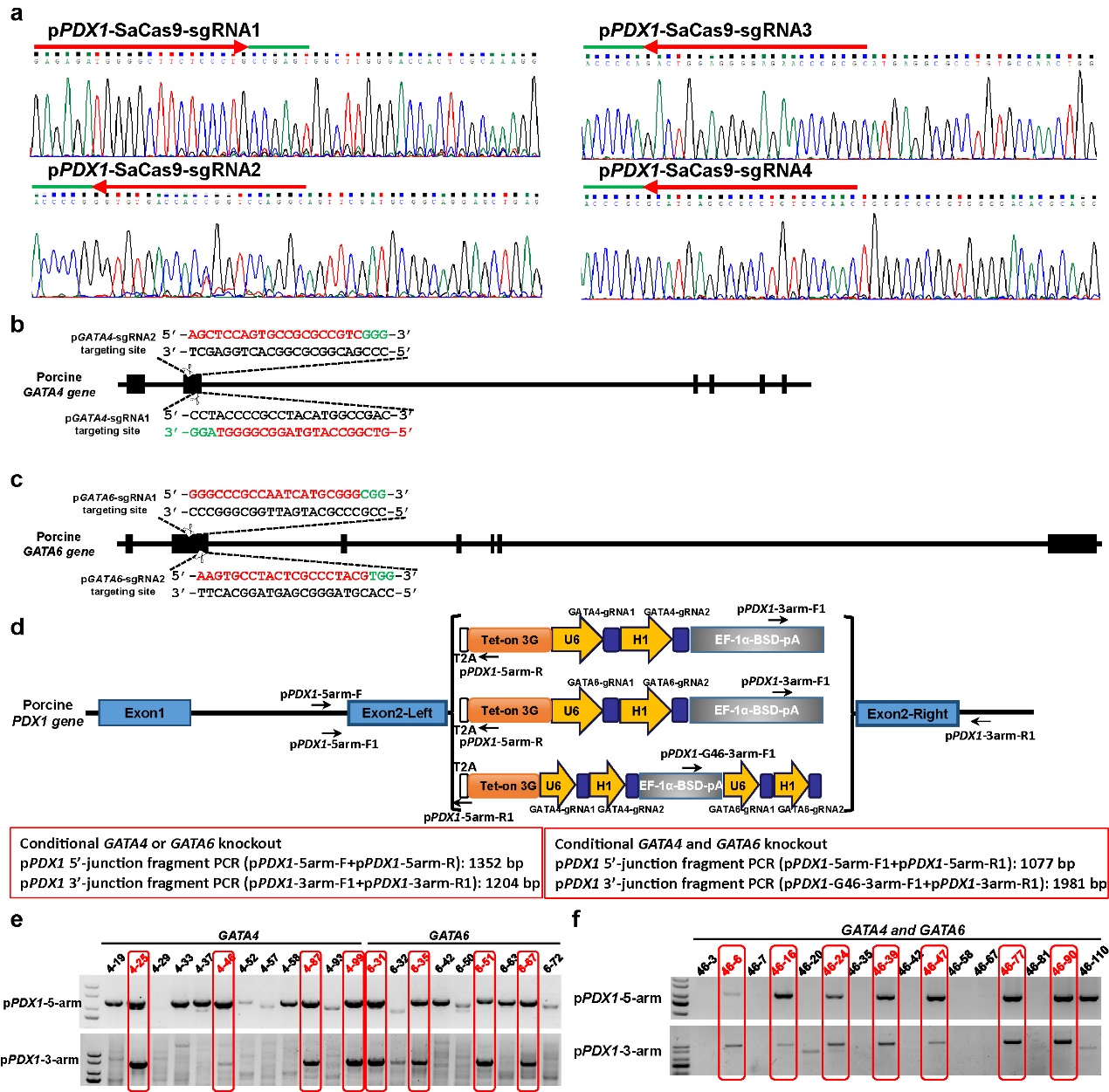
**

**Fig. S6. SaCas9-mediated knock-in of spatiotemporal gene knockout cassette into the porcine *PDX1* locus.**

**a** Sanger sequencing for evaluating the effects of different SaCas9-sgRNAs targeting porcine *PDX1* locus. **b** Diagram of sgRNAs to knockout porcine *GATA4*. **c** Diagram of sgRNAs to knockout porcine *GATA6*. **d.** Scheme for the integration of 2A-rtTA-polyA cassette and U6-sgRNA-expressing cassette at the upstream of the stop codon of the porcine *PDX1* gene. PCR primers used for genotyping were labeled as black arrowheads. **e-f** Representative genotyping PCR analysis to confirm the correct homologous recombination at the porcine *PDX1* locus in PFFs carrying TRE3G-controlled Cas9 expression cassette at the *Hipp11* locus. The 5′- and 3′-junction fragment PCR showed 4-25, 4-46, 4-87, and 4-99 cell colony carrying the correct transgene integration for spatiotemporal knockout of *GATA4* (**e, left panel**), 6-31, 6-35, 6-51, and 6-67 cell colony for spatiotemporal knockout of *GATA6* (**e, right panel**), and 46-6, 46-16, 46-24, 46-39, 46-47, 46-77, and 46-90 cell colony for simultaneously spatiotemporal knockout of *GATA4* and *GATA6* (**f**).

**
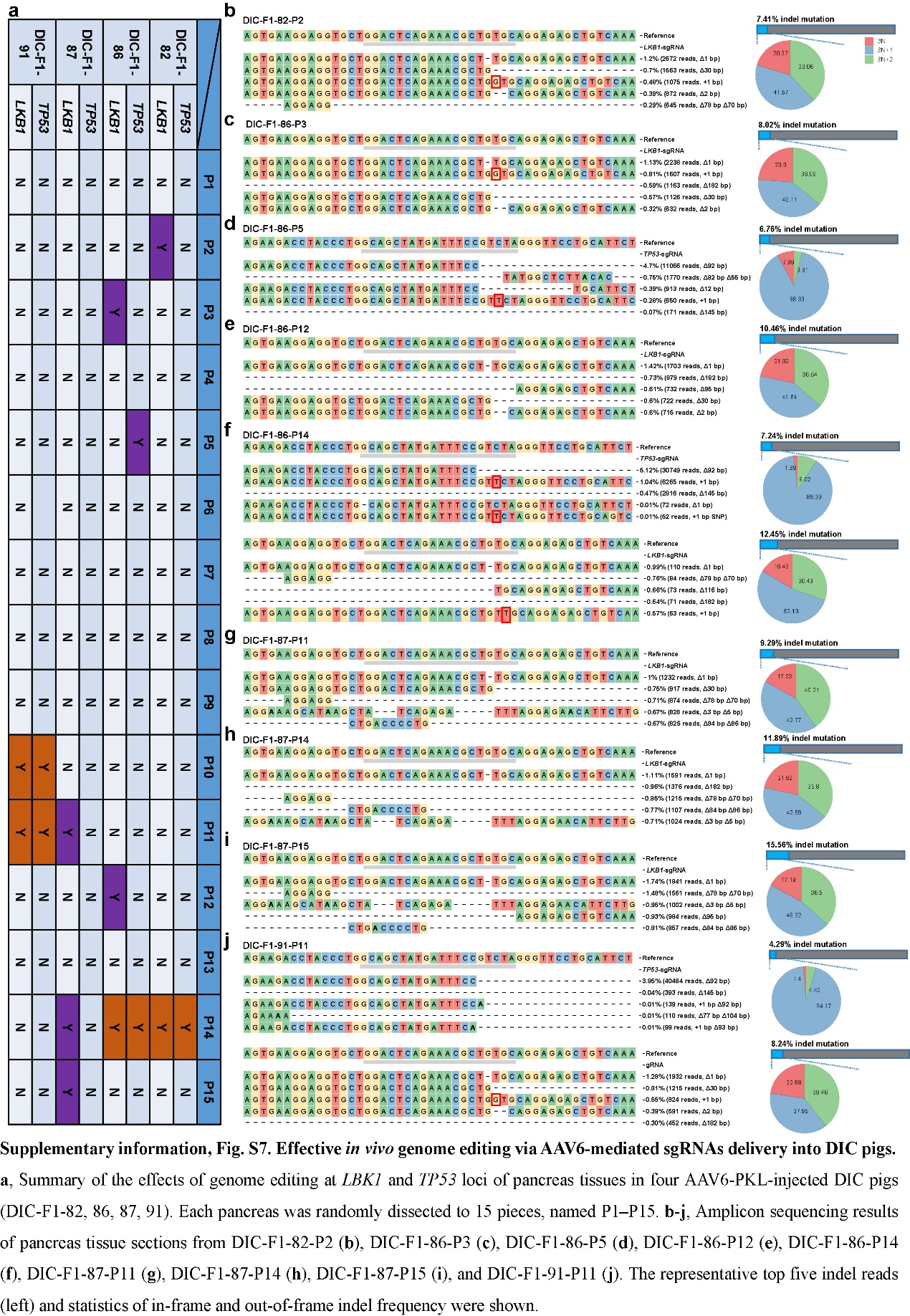
**

**Fig. S7. Effective *in vivo* genome editing via AAV6-mediated sgRNAs delivery into DIC pigs.**

**a** Summary of the effects of genome editing at *LBK1* and *TP53* loci of pancreas tissues in four AAV6-PKL-injected DIC pigs (DIC-F1-82, 86, 87, 91). Each pancreas was randomly dissected to 15 pieces, named P1**–**P15. **b-j** Amplicon sequencing results of pancreas tissue sections from DIC-F1-82-P2 (**b**), DIC-F1-86-P3 (**c**), DIC-F1-86-P5 (**d**), DIC-F1-86-P12 (**e**), DIC-F1-86-P14 (**f**), DIC-F1-87-P11 (**g**), DIC-F1-87-P14 (**h**), DIC-F1-87-P15 (**i**), and DIC-F1-91-P11 (**j**). The representative top five indel reads (left) and statistics of in-frame and out-of-frame indel frequency were shown.


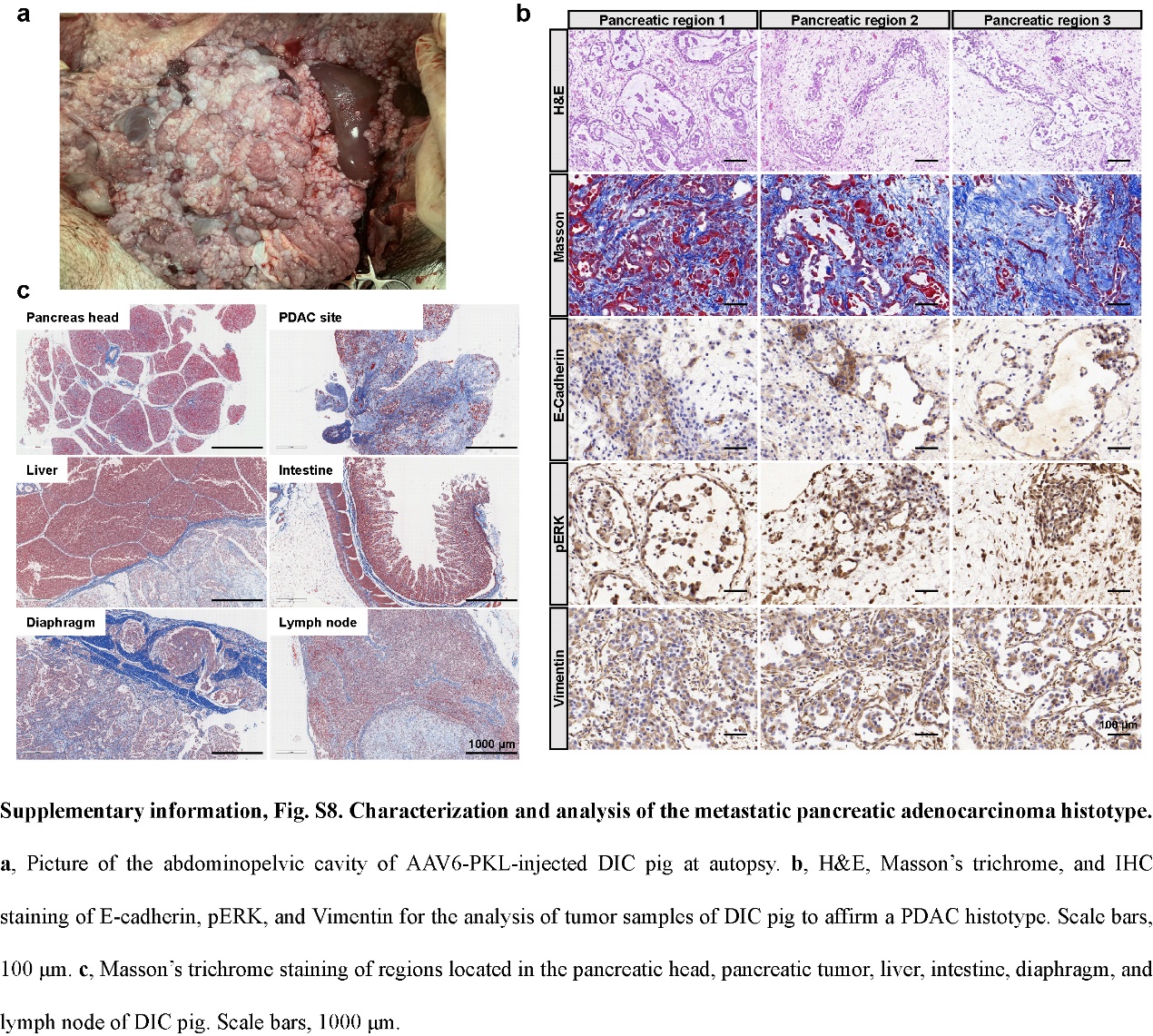


**Fig. S8. Characterization and analysis of the metastatic pancreatic adenocarcinoma histotype.**

**a** Picture of the abdominopelvic cavity of AAV6-PKL-injected DIC pig at autopsy. **b** H&E, Masson’s trichrome, and IHC staining of E-cadherin, pERK, and Vimentin for the analysis of tumor samples of DIC pig to affirm a PDAC histotype. Scale bars, 100 μm. **c** Masson’s trichrome staining of regions located in the pancreatic head, pancreatic tumor, liver, intestine, diaphragm, and lymph node of DIC pig. Scale bars, 1000 μm.

**.**
