# Supplementary material for: Doxycycline-dependent Cas9-expressing pig resources for conditional in vivo gene nullification and activation
Source: Genome Biol. 2023 Jan 17;24:8. doi: 10.1186/s13059-023-02851-x (PMC9843877; doi:10.1186/s13059-023-02851-x)
Supplement: Supplementary file 2 — Additional file 2. Table S1 for Doxycycline-dependent Cas9-expressing pig resources for conditional in vivo gene nullification and activation. [file 13059_2023_2851_MOESM2_ESM.docx]

**Table S1. Summary of F1 pigs.**
