# Supplementary material for: Doxycycline-dependent Cas9-expressing pig resources for conditional in vivo gene nullification and activation
Source: Genome Biol. 2023 Jan 17;24:8. doi: 10.1186/s13059-023-02851-x (PMC9843877; doi:10.1186/s13059-023-02851-x)
Supplement: Supplementary file 3 — Additional file 3. Table S2 for Doxycycline-dependent Cas9-expressing pig resources for conditional in vivo gene nullification and activation. [file 13059_2023_2851_MOESM3_ESM.docx]

| **Primers** | **Sequences (5′-3′)** |
| --- | --- |
| **Genome editing primers** | |
| ***PCSK9*-sgRNA-F** | CACCGGTGCTCGGCTTCAGGTCGG |
| ***PCSK9*-sgRNA-R** | AAACCCGACCTGAAGCCGAGCACC |
| ***ALK*-sgRNA-F** | CACCGGATTAGAACACAAGTCCTC |
| ***ALK*-sgRNA-R** | AAACGAGGACTTGTGTTCTAATCC |
| ***EML4*-sgRNA-F** | CACCGTGAAGTGCCAGAGCATACA |
| ***EML4*-sgRNA-R** | AAACTGTATGCTCTGGCACTTCAC |
| ***OCT4*-sgRNA-F** | CACCGCGCACCTCAGGTCGGAGTG |
| ***OCT4*-sgRNA-R** | AAACCACTCCGACCTGAGGTGCGC |
| ***LMNA*-sgRNA-F** | CACCGGCATCAAGTCTGCCTACG |
| ***LMNA*-sgRNA-R** | AAACCGTAGGCAGACTTGATGCC |
| ***TP53*-sgRNA-F** | CACCGCAGCTATGATTTCCGTCTA |
| ***TP53*-sgRNA-R** | AAACTAGACGGAAATCATAGCTGC |
| ***KRAS*-sgRNA-F** | CACCGTAGTTGGAGCTGGTGGCGT |
| ***KRAS*-sgRNA-F** | AAACACGCCACCAGCTCCAACTAC |
| ***APC*-sgRNA-F** | CACCGGCAACTTCGGGTAACGGTC |
| ***APC*-sgRNA-R** | AAACGACCGTTACCCGAAGTTGCC |
| ***LKB1*-sgRNA-F** | CACCGGACTCAGAAACGCTGTGC |
| ***LKB1*-sgRNA-R** | AAACGCACAGCGTTTCTGAGTCC |
| **Epigenomic editing primers** | |
| ***CDX2*-dgRNA1-F** | CACCGTCCCCAGGCAGCA |
| ***CDX2*-dgRNA1-R** | AAACTGCTGCCTGGGGAC |
| ***CDX2*-dgRNA2-F** | CACCGCAGCCTCCAGCGT |
| ***CDX2*-dgRNA2-R** | AAACACGCTGGAGGCTGC |
| ***CDX2*-dgRNA3-F** | CACCGGGGAAGGGGCGAG |
| ***CDX2*-dgRNA3-R** | AAACCTCGCCCCTTCCCC |
| ***CDX2*-dgRNA4-F** | CACCGCAGCAGCGCGCTC |
| ***CDX2*-dgRNA4-R** | AAACGAGCGCGCTGCTGC |
| ***CDX2*-dgRNA5-F** | CACCGCGGTCCCTCCCTC |
| ***CDX2*-dgRNA5-R** | AAACGAGGGAGGGACCGC |
| ***CDX2*-dgRNA6-F** | CACCGAAGGAAGAAAGAG |
| ***CDX2*-dgRNA6-R** | AAACCTCTTTCTTCCTTC |
| ***SOX2*-dgRNA1-F** | CACCGCTGTGCGCGGGCC |
| ***SOX2*-dgRNA1-R** | AAACGGCCCGCGCACAGC |
| ***SOX2*-dgRNA2-F** | CACCGGGTCGGCTGCTGC |
| ***SOX2*-dgRNA2-R** | AAACGCAGCAGCCGACCC |
| ***SOX2*-dgRNA3-F** | CACCGGCCGGGACTTTGG |
| ***SOX2*-dgRNA3-R** | AAACCCAAAGTCCCGGCC |
| ***SOX2*-dgRNA4-F** | CACCGAGGAGAGGCGGGC |
| ***SOX2*-dgRNA4-R** | AAACGCCCGCCTCTCCTC |
| ***SOX2*-dgRNA5-F** | CACCGTCTGATTTTCCTCG |
| ***SOX2*-dgRNA5-R** | AAACCGAGGAAAATCAGAC |
| ***SOX2*-dgRNA6-F** | CACCGGCAAACTGGAATC |
| ***SOX2*-dgRNA6-R** | AAACGATTCCAGTTTGCC |
| **Spatio-temporal gene knockout primers** | |
| ***PDX1*-ASgRNA1-F** | AGATCTGTAATACCGGGGCTGCTTCTG |
| ***PDX1*-ASgRNA1-R** | GATACAGAAGCAGCCCCGGTATTACAG |
| ***PDX1*-ASgRNA2-F** | AGATGCCTCTCTAAAGAGGTCCACGGT |
| ***PDX1*-ASgRNA2-R** | GATAACCGTGGACCTCTTTAGAGAGGC |
| ***PDX1*-ASgRNA3-F** | AGATGAGAGGCCAAATCTTCAAGACAG |
| ***PDX1*-ASgRNA3-R** | GATACTGTCTTGAAGATTTGGCCTCTC |
| ***PDX1*-SagRNA1-F** | CACCGAGAGATGGGGCTTCTCCCTG |
| ***PDX1*-SagRNA1-R** | AAACCAGGGAGAAGCCCCATCTCTC |
| ***PDX1*-SagRNA2-F** | CACCGCCTGGACCGGTGGTCACACC |
| ***PDX1*-SagRNA2-R** | AAACGGTGTGACCACCGGTCCAGGC |
| ***PDX1*-SagRNA3-F** | CACCGCGCGGGTTCTCCCCTCCAGTC |
| ***PDX1*-SagRNA3-R** | AAACGACTGGAGGGGAGAACCCGCGC |
| ***PDX1*-SagRNA4-F** | CACCGTTGGCACAGGCGCCTCATGC |
| ***PDX1*-SagRNA4-R** | AAACGCATGAGGCGCCTGTGCCAAC |
| ***GATA4*-sgRNA1-F** | CACCGTCGGCCATGTAGGCGGGGT |
| ***GATA4*-sgRNA1-R** | AAACACCCCGCCTACATGGCCGAC |
| ***GATA4*-sgRNA2-F** | TCCCAGCTCCAGTGCCGCGCCGTC |
| ***GATA4*-sgRNA2-R** | AAACGACGGCGCGGCACTGGAGCT |
| ***GATA6*-sgRNA1-F** | CACCGGGCCCGCCAATCATGCGGG |
| ***GATA6*-sgRNA1-R** | AAACCCCGCATGATTGGCGGGCCC |
| ***GATA6*-sgRNA2-F** | TCCCAAGTGCCTACTCGCCCTACG |
| ***GATA6*-sgRNA2-R** | AAACCGTAGGGCGAGTAGGCACTT |
| **Q-PCR primers** | |
| **Q-h*KRAS*^G12D^-F** | ACTTGTGGTAGTTGGAGCTGA |
| **Q-h*KRAS*^G12D^-R** | TTGGATCATATTCGTCCACAA |
| **Q-p*SOX2*-F** | GTTCCATGGGCTCAGTGGTCAAG |
| **Q-p*SOX2*-R** | AAGCGTACCGGGTTTTTCTCCATAC |
| **Q-p*CDX2*-F** | GTCGCTACATCACCATTCGG |
| **Q-p*CDX2*-R** | GATTTTCCTCTCCTTCGCTCT |
| **Q-p*ACTB*-F** | TGAACCCCAAAGCCAACCGTG |
| **Q-p*ACTB*-R** | TGTAGCCCCGCTCCGTCAGGA |
| **Q-tdtomato-F** | GCTGAAGGGCGAGATCCA |
| **Q-tdtomato-R** | GTGGGAGGTGATGTCCAGCTT |
| **Q-p*GAPDH*-F** | ACCTGCCGCCTGGAGAAACC |
| **Q-p*GAPDH*-R** | GACCATGAGGTCCACCACCCTG |
| **PCR primers** | |
| ***PCSK9*-det-F** | TTGGGAAGAGGGCCAAGCCA |
| ***PCSK9*-det-R** | TAAACCGAGGACGGAGAGGTG |
| ***LKB1*-det-F** | CAGCAGCTGGGCATGTTTAC |
| ***LKB1*-det-R1** | CAACAAGCTAGGCTGACTTC |
| **p*LKB1*-det-R** | TTCTTCACATTGGCCTCCCC |
| ***EML4*-F** | TGGGGAATGGAGATGTGCTT |
| ***ALK*-R** | TGAGGGTGATGTTTTTCCGAG |
| **pig-*β-actin*-F** | GCCAACCGTGAGAAGATGAC |
| **pig-*β-actin*-R** | GAGTCCATCACGATGCCAGT |
| **H11-det-F** | GCGAGAATTCTAAACTGGAG |
| **H11-det-R** | CTCAAGCTCCTCAATTCACC |
| ***APC*-F** | GCTCCATTAAATGCCAGAGCCA |
| ***APC*-R** | CCGATTGTTCTGGAGATACCCA |
| ***KRAS*-F** | GCACATCTGTGGTCAACGGGC |
| ***KRAS*-R** | CTCCCCAGAGAAGACTGAAGAC |
| ***TP53*-F** | TCCATCCGCAGTCCTCTGAGCT |
| ***TP53*-R** | GATGAGAGGCCAAGGTCAAGTG |
| **A** | GCCCCCTCCCCATGAATCAT |
| **B** | TAGGGGCCAAAGTCAGCCATC |
| **C** | GCCTTACTCCTGCTCAAGCA |
| **D** | CCAACACCACACAGTTAGCTAG |
| ***OCT4*-det-F** | TTGGAGAGCCCTGGTTTTAC |
| ***OCT4*-det-R** | TTGTCTCTAAATTCGGCGCC |
| ***LMNA*-det-F** | ATCACCCGGCTACAGGAGAA |
| ***LMNA*-det-R** | TGCCTTGAGCTCCTTGAACT |
| **p*PDX1*-5arm-F** | TACAGAGGATCTACCAGCTC |
| **p*PDX1*-5arm-R** | TGTTCTTCACGTGCCAGTAC |
| **p*PDX1*-3arm-F1** | AGGACTGACACTCGACCTCG |
| **p*PDX1*-3arm-R1** | AGGATGGCAGCTCTGGTCAG |
| **p*PDX1*-5arm-F1** | CAGCTCAAGGTTTTTCCAGC |
| **p*PDX1*-5arm-R1** | GGGATTCTCCTCCACGTCAC |
| **p*PDX1*-G46-3arm-F1** | TTATGTGTGGGAGGGCTAAG |
| **R5-F** | TGCGTGAGTCTCTGAGCGCAG |
| **R5-R** | CATCAAGGAAACCCTGGACTACTG |
| **R3-F** | CATCGCATCGAGCGAGCACGTA |
| **R3-R** | CAACTCAGTGTGACTTGAGCAG |
| **R-F** | CTCGTCATCGCCTCCATGTCAG |
| **R-R** | GTTGGGCCTATGCTCAAGATGG |
| **rtTA-R** | CAGGCCTTCGATACCGACTCC |
| **H5-F** | GGTGTCTTCACCAAGAGGTGAC |
| **H5-R** | ACTCGAGTGAAGACGAAAGGGC |
| **H3-F** | GACAATAGCAGGCATGCTGGG |
| **H3-R** | GGACCTGACTTCCCTTCCGTCT |
| **H-F** | GTTCAGGCTGGGCTGACCCTATA |
| **H-R** | GGATTGGGATTCAGGTCTCTGCT |

**Table S2. Primers used in this study.**
