# Supplementary material for: Doxycycline-dependent Cas9-expressing pig resources for conditional in vivo gene nullification and activation
Source: Genome Biol. 2023 Jan 17;24:8. doi: 10.1186/s13059-023-02851-x (PMC9843877; doi:10.1186/s13059-023-02851-x)
Supplement: Supplementary file 4 — Additional file 4. Review history. [file 13059_2023_2851_MOESM4_ESM.docx]

Review History

**First round of review**

**Reviewer 1**

**Are you able to assess all statistics in the manuscript, including the appropriateness of statistical tests used?** There are no statistics in the manuscript.

**Comments to author:**

Jin et al. construct the novel doxycycline-inducible SpCas9-expressing (DIC) pig resources for facile in vivo genome and epigenome modification in large animal model. Compared with mouse models, large animal models can be used as better disease models because they are more similar to humans, but the generation of the specific gene-editing large animal models is more complex, costly and time-consuming. When sgRNAs targeting specific genes (e.g., TP53, LKB1, ALK, EML4, etc.) are successfully delivered, the DIC pigs which have carried constitutive/tissue specific promoter-driving Tet-on 3G and TRE3G-driving SpCas9-expressing cassette, could be used to mimic the specific cancer under the in vivo spatiotemporal control via precise chemical induction. The porcine DIC systems overcome the in vivo delivery challenge of Cas9 protein and avoid the side effects of constitutive Cas9 expression in pig models, which will empower a wide range of agricultural, biological, and disease modeling applications. The work of this paper is original and logical. However, there are some problems to be further improved as well.

-------------------------------------------------------------------------------------------
Some amendments are suggested in the following points.
Major points:
- Given that the somatic cells (1#, 14#, 23#, 24#, and 29#) have been identified for TRE3G-driving SpCas9-T2A-Tdtomato-expressing cassette integration at pHipp11 loci, why did half of cloned piglets not contain this cassette integration?
- Different injected dosages of AAV6-PKL were chosen for the DIC-F1 pigs, which did not seem to be calculated by body weight of different DIC-F1 pigs. Why did the authors choose this design and whether there are potential effects of different dosages?
Minor points:
- How to identify homozygosity or heterozygosity of fragment integration at pRosa26 and pHipp11 locus by PCR is confusing. Please mark the position of primers in figures clearly.

- The number of cloned piglets (1106-1#, 1106-3#, and 1106-5#) should be changed to 0101-1#, 0101-3#, and 0101-5#.

- In Fig. 3d-e, please clearly mark different treatment groups (e.g., pre-tRNA-linked ALK- and EML4-sgRNAs).
- In Fig. S3, the IHC staining results of untreated DIC controls should be added.
- Please check and revise the layout of full manuscripts. Many of figure sequences are not consistent between the results section and the figure section.

**Reviewer 2**

**Are you able to assess all statistics in the manuscript, including the appropriateness of statistical tests used?** No, I do not feel adequately qualified to assess the statistics.

**Comments to author:**

Jin et al. present a new dox-inducible SpCas9-expressing pig model that was generated by knock-in of the binary tetracycline-inducible elements into the ROSA26 and Hipp11 loci. They validated the feasibility of in vivo gene mutation by inducing primary and metastatic pancreatic ductal adenocarcinoma after delivery of a single AAV6 vector containing TP53- and LKB1-sgRNA and a mutant human KRAS gene into the adult pancreas. In a proof-of-concept trial for validation of the application, they achieved effective in vivo gene editing and rapidly established a metastatic PDAC pig model. The manuscript is well written, the results are convincing and I would like to congratulate the authors for the generation of this important pig model. There are only a few minor points that should be addressed:
Abstract: The authors speak of over-expression of the binary tetracycline-inducible elements. These elements are naturally expressed in the pig, therefore you can not speak of an over-expression. Change that to expression
Background p6, L52: The authors should be consistent with the spelling of modelling, sometimes they use modeling and in other sentences modelling. Please check and change to modelling.
As the authors mentioned that expression of Cre-recombinase was confirmed to negatively affect porcine embryonic development, they should also discuss their results in that context. The overall cloning efficiency is not very high <1% and they also observed perinatal death.

**Authors Response**

**Point-by-point responses to the reviewers’ comments:**

**Reviewer 1:**

***Jin et al. construct the novel doxycycline-inducible SpCas9-expressing (DIC) pig resources for facile in vivo genome and epigenome modification in large animal model. Compared with mouse models, large animal models can be used as better disease models because they are more similar to humans, but the generation of the specific gene-editing large animal models is more complex, costly and time-consuming. When sgRNAs targeting specific genes (e.g., TP53, LKB1, ALK, EML4, etc.) are successfully delivered, the DIC pigs which have carried constitutive/tissue specific promoter-driving Tet-on 3G and TRE3G-driving SpCas9-expressing cassette, could be used to mimic the specific cancer under the in vivo spatiotemporal control via precise chemical induction. The porcine DIC systems overcome the in vivo delivery challenge of Cas9 protein and avoid the side effects of constitutive Cas9 expression in pig models, which will empower a wide range of agricultural, biological, and disease modeling applications. The work of this paper is original and logical. However, there are some problems to be further improved as well.***

**Response:** We thank the reviewer for the positive assessment of our manuscript and the helpful comments.

**Major points:**

***1. Given that the somatic cells (1#, 14#, 23#, 24#, and 29#) have been identified for TRE3G-driving SpCas9-T2A-Tdtomato-expressing cassette integration at pHipp11 loci, why did half of cloned piglets not contain this cassette integration?***

**Response**: Thanks a lot for your careful reading. In theory, the cell colony we picked and identified was from one single cell with a specific genotype. However, two reasons might lead to positive colonies mixed with other cells. First, when we simultaneously trypsinized and resuspended different colonies within one 10 cm dish using cloning cylinders, cells without precise integration of TRE3G-driving SpCas9-T2A-Tdtomato-expressing cassette may be intermixed with positive colonies because of liquid leakage from different cloning cylinders. Second, the picked colonies might derive from several cells including both positive and negative cells, although we plated the transfected porcine fibroblasts (PFFs) at limiting dilutions for clonal isolation. Thus, it was possible to generate cloned piglets that not contain this cassette integration at the p*Hipp11* locus when these impure and pooled positive colonies were used as donor cells for somatic cell nuclear transfer cloning.

***2. Different injected dosages of AAV6-PKL were chosen for the DIC-F1 pigs, which did not seem to be calculated by body weight of different DIC-F1 pigs. Why did the authors choose this design and whether there are potential effects of different dosages?***

**Response**: Thanks for your kindly questions. Unlike systemic virus delivery by intravenous or intraperitoneal injection, where injected dosages depend on animal weight, here we adopted the local delivery of viruses into the main pancreatic duct and/or the body of the pancreas. We referred to the recommended infusion parameters of mouse models in published literature to determine the range of virus injection dosage of DIC-F1 pigs [1, 2]. The size of the pancreas in different pigs does not always positively correlate with the weight of the pig. Thus, different injected dosages of AAV6-PKL were chosen here for the DIC-F1 pigs, not only calculated by body weight of different DIC-F1 pigs, but also depended on pancreas size, main pancreatic duct or the body of the pancreas injection. In theory, a higher injection dosage may lead to more efficient sgRNA delivery, resulting in higher gene editing efficiency.

**Minor points:**

***1.*** ***How to identify homozygosity or heterozygosity of fragment integration at pRosa26 and pHipp11 locus by PCR is confusing. Please mark the position of primers in figures clearly.***

**Response**: Thanks for the comment. In this study, we used competitive PCR with three primers to identify homozygosity or heterozygosity of fragment integration at p*Rosa26* and p*Hipp11* loci. For these three primers, two were located at the sides of the targeting sites and one was located at the exogenous sequences. For wild-type and homozygous piglets, only one PCR band (wild-type: 590 bp at the p*Rosa26* locus and 892 bp at the p*Hipp11* locus; precise knock-in: 380 bp at the p*Rosa26* locus and 556 bp at the p*Hipp11* locus) could be amplified. For heterozygous piglets, both wild-type and knock-in fragment could be amplified. We re-organized the **Fig. S1** and marked the position of primers in **Fig. S1a, b** in the revised manuscript as suggested.

**a** Scheme for the rtTA-expressing cassette inserted into the p*Rosa26* locus. **b** Scheme for the integration of TRE3G-driving SpCas9-T2A-tdTomato-expressing cassette into the p*Hipp11* locus via CRISPR/Cpf1-mediated HDR. In **Fig. S1a** and **b**, PCR primers used for knock-in identification and homozygote or heterozygote identification were indciated by black and red arrowheads, respectively.

***2. The number of cloned piglets (1106-1#, 1106-3#, and 1106-5#) should be changed to 0101-1#, 0101-3#, and 0101-5#.***

**Response**: Thanks for the kind remind. We have corrected in the revised manuscript.

***3. In Fig. 3d-e, please clearly mark different treatment groups (e.g., pre-tRNA-linked ALK- and EML4-sgRNAs).***

**Response**: Thanks for the suggestion. We re-annotated the different treatment groups as suggested and added the following figure legends in the revised manuscripts: “In **Fig. d** and **e**, “+1” group refers to the simultaneous transfection of two plasmids containing *ALK*-sgRNA and *EML4*-sgRNA, respectively; “+2” group refers to transfection of a single plasmid containing pre-tRNA-linked *ALK*- and *EML4*-sgRNAs.”

***4. In Fig. S3, the IHC staining results of untreated DIC controls should be added.***

**Response**: We added the IHC staining results of untreated DIC controls in **Fig. S3** as reviewer suggested.

**Fig. S3. IHC staining results for comparison of SpCas9 expression induced by different Dox administration methods in multiple organs from adult DIC pigs. Scale bars, 100 μm.**

***5. Please check and revise the layout of full manuscripts. Many of figure sequences are not consistent between the results section and the figure section.***

**Response**: We appreciated the kind remind. We checked and revised the layout of full manuscripts to make sure that the figure sequences are consistent between the Results section and the Figure section.

**Reviewer 2:**

***Jin et al. present a new dox-inducible SpCas9-expressing pig model that was generated by knock-in of the binary tetracycline-inducible elements into the ROSA26 and Hipp11 loci. They validated the feasibility of in vivo gene mutation by inducing primary and metastatic pancreatic ductal adenocarcinoma after delivery of a single AAV6 vector containing TP53- and LKB1-sgRNA and a mutant human KRAS gene into the adult pancreas. In a proof-of-concept trial for validation of the application, they achieved effective in vivo gene editing and rapidly established a metastatic PDAC pig model. The manuscript is well written, the results are convincing and I would like to congratulate the authors for the generation of this important pig model. There are only a few minor points that should be addressed:***

**Response:** We thank the reviewer for the positive comments to our work.

***1. Abstract: The authors speak of over-expression of the binary tetracycline-inducible elements. These elements are naturally expressed in the pig, therefore you can not speak of an over-expression. Change that to expression.***

**Response**: We have corrected the “over-expression” to “expression” in the revised manuscript as suggested.

***2. Background p6, L52: The authors should be consistent with the spelling of modelling, sometimes they use modeling and in other sentences modelling. Please check and change to modelling.***

**Response**: Thanks for your kind remind. We checked and changed “modeling” to “modelling” in the revised manuscript.

***3. As the authors mentioned that expression of Cre-recombinase was confirmed to negatively affect porcine embryonic development, they should also discuss their results in that context. The overall cloning efficiency is not very high <1% and they also observed perinatal death.***

**Response**: Thanks for the comment to improve the quality of our manuscript. To address the question of the reviewer, we added following sentences in the Results section of the revised manuscript.

“When reconstructed embryos were cultured for 6 days, almost no difference of blastocyst rates between Dox-treated (19.5%, 40/205) and Dox-untreated (20.4%, 23/113) groups was observed, while the previous report showed that zygote injection of RNA encoding Cre recombinase resulted in reduction of blastocyst rate [26], indicating that Dox-dependent SpCas9 expression may have less negative impact on porcine embryo development than Cre recombinase expression.”

“The low cloning efficiency and the perinatal death might be due to overall inefficient SCNT procedures as that previously reported [32], rather than genetic modification of donor cells per se.”

**References**

1.	Guo P, Wiersch J, Xiao X, Gittes G: **Simplified Purification of AAV and Delivery to the Pancreas by Intraductal Administration.** *Methods in molecular biology (Clifton, NJ)* 2019, **1950:**373-387.

2.	Quirin KA, Kwon JJ, Alioufi A, Factora T, Temm CJ, Jacobsen M, Sandusky GE, Shontz K, Chicoine LG, Clark KR, et al: **Safety and Efficacy of AAV Retrograde Pancreatic Ductal Gene Delivery in Normal and Pancreatic Cancer Mice.** *Molecular Therapy-Methods & Clinical Development* 2018, **8:**8-20.

**Second round of review**

**Reviewer 1**

I am satisfied with the revision and recommend for publication.
